# Intensive Systolic Blood Pressure Reduction and Kidney and Cardiovascular Outcomes: A Secondary Analysis of a Randomized Clinical Trial

**DOI:** 10.1001/jamanetworkopen.2025.19604

**Published:** 2025-07-11

**Authors:** Guozhe Sun, Wei Miao, Songyue Liu, Yangzhi Yin, Danxi Geng, Ning Ye, Ziyi Xie, Linlin Zhang, Shiyu Zhou, Chang Wang, Lixia Qiao, Sitong Pei, Nanxiang Ouyang, Chuning Shi, Xiaofan Guo, Yingxian Sun

**Affiliations:** 1 Department of Cardiology, The First Hospital of China Medical University, Shenyang, Liaoning, China; 2 Department of Clinical Epidemiology and Evidence-Based Medicine, The First Hospital of China Medical University, Shenyang, China

## Abstract

**Importance:**

Intensive blood pressure (BP) control is effective in reducing major cardiovascular events. However, whether the reduction of systolic BP may lead to kidney injury remains controversial.

**Objective:**

To elucidate the association of intensive BP control with kidney and cardiovascular outcomes in individuals with hypertension without chronic kidney disease.

**Design, Setting, and Participants:**

This study is a secondary analysis of the China Rural Hypertension Control Project (CRHCP), an open-label, blind-end-point cluster randomized trial conducted from May 8, 2018, to March 15, 2023, in 326 villages in China. Patients with a baseline estimated glomerular filtration rate (eGFR) of 60 mL/min/1.73 m^2^ or greater were randomized to an intervention or usual care group, stratified according to eGFR level.

**Exposures:**

A nonphysician community health care professional implemented a multifaceted intervention program after training to achieve a BP treatment goal of less than 130/80 mm Hg.

**Main Outcomes and Measures:**

Kidney outcome was defined as an eGFR decrease of 30% to a rate less than 60 mL/min/1.73 m^2^. Composite cardiovascular outcomes included cardiovascular death, stroke, myocardial infarction, and heart failure.

**Results:**

A total of 33 332 patients with a baseline eGFR of 60 mL/min/1.73 m^2^ or greater were included in this subgroup analysis (mean [SD] age, 62.8 [9.1] years; 61.3% female). After 36 months of follow-up, in 7562 participants with eGFRs between 60 and 90 mL/min/1.73 m^2^, 121 participants in the intervention group and 101 in the usual care group (risk ratio, 1.17; 95% CI, 0.82-1.62; *P* = .36) experienced kidney outcome, which was not statistically significant. As for cardiovascular outcomes, 210 participants (2.0% per year) in the intervention group and 346 participants (3.3% per year) in the usual care group had the composite cardiovascular disease outcome, and the disparity between the 2 groups was statistically significant (hazard ratio, 0.57; 95% CI, 0.47-0.68; *P* < .001).

**Conclusions and Relevance:**

This secondary analysis of the CRHCP study suggests that intensive BP control was associated with reduced incidence of the composite cardiovascular disease outcome but not increased risk of kidney injury in patients without chronic kidney disease. These findings provide further evidence supporting the implementation of intensive BP control in patients without chronic kidney disease.

**Trial Registration:**

ClinicalTrials.gov Identifier: NCT03527719

## Key Points

**Question:**

Is the reduction of systolic blood pressure associated with kidney injury and cardiovascular diseases?

**Findings:**

This secondary analysis of 7562 China Rural Hypertension Control Project participants with estimated glomerular filtration rates between 60 and 90 mL/min/1.73 m^2^ found no significant difference in kidney outcomes between the intervention and usual care groups. However, the intervention group had a significantly lower rate of composite cardiovascular diseases compared with the usual care group.

**Meaning:**

These findings suggest that intensive blood pressure control reduced the incidence of composite cardiovascular disease without increasing the risk of kidney injury in patients without chronic kidney disease.

## Teaser

This secondary analysis of the China Rural Hypertension Control Project examines the association of intensive blood pressure control with kidney and cardiovascular outcomes in individuals with hypertension without chronic kidney disease.

## Introduction

The global burden of hypertension, which affects 1.28 billion adults worldwide, poses a substantial public health challenge.^1^ Hypertension is one of the most important risk factors for cardiovascular diseases (CVDs) and chronic kidney injury.^2,3,4^ The dysfunction caused by chronic kidney injury is irreversible.^5,6^ Therefore, the investigation of the association between blood pressure (BP) control and kidney function is crucial.

Intensive BP control has been demonstrated to additionally reduce major cardiovascular events in patients with hypertension compared with traditional BP targets.^7,8,9,10,11^ The Systolic Blood Pressure Intervention Trial (SPRINT) highlighted a reduction in cardiovascular outcomes on achieving a systolic BP (SBP) level below 120 mm Hg.^10^ The 2017 American College of Cardiology/American Heart Association guidelines recommended intensive BP lowering to an SBP level below 130 mm Hg and noted the need for implementation studies to demonstrate the benefits and safety of intensive BP control in resource-constrained practice settings.^12^

However, when implementing intensive BP control, the risk of kidney injury should be taken into consideration. A substantial reduction of SBP levels may impair the kidney function. A meta-analysis study revealed that SBP lower than 120 mm Hg results in loss of kidney protection and a SBP less than 110 mm Hg increases kidney injury.^13^ A subgroup analysis of the SPRINT study showed that a SBP less than 120 mm Hg increased 2.6% of kidney outcomes compared with a SBP less than 140 mm Hg in individuals without chronic kidney disease (CKD), yet this finding was outweighed by the potential benefits for cardiovascular and all-cause mortality.^14^ Similarly, a secondary analysis of 2 randomized clinical trials, including SPRINT and Action to Control Cardiovascular Risk in Diabetes (ACCORD), showed that intensive BP control also increased the risk of kidney outcomes in populations with and without type 2 diabetes.^15^ In the Strategy of Blood Pressure Intervention in the Elderly Hypertensive Patients (STEP) trial,^8^ intensive BP control in the elderly population did not increase the risk of kidney outcomes but reduced the risk of cardiovascular outcomes. Therefore, the effect of intensive BP control on kidney outcomes seems to be controversial because all these previous studies were conducted in special populations. Therefore, there is an urgent need for studies involving more representative populations to provide robust evidence on whether intensive BP control increases the risk of kidney outcomes in patients without CKD.

The China Rural Hypertension Control Project (CRHCP) was designed to highlight the effectiveness and safety of an intensive BP strategy (<130/80 mm Hg) led by nonphysician community health care professionals.^9,16^ In this secondary analysis, we examined the association of intensive BP control with kidney and cardiovascular outcomes in individuals with hypertension without CKD.

## Methods

### Study Population

This secondary analysis of the CRHCP aims to identify the association of intensive BP control with kidney injury. The CRHCP was an open-label, blind-end-point cluster randomized trial conducted in 326 villages of rural China from May 8, 2018, to March 15, 2023.^9,16^ For this secondary analysis, we analyzed the data from the 36-month follow-up of the CRHCP. Specific study protocol details for the CRHCP have been published previously.^17^ All participants provided written informed consent on enrollment in the trial. The original study was approved by the ethics committees of all participating institutions. The ethics committees waived the requirement for ethical approval for this secondary analysis. This secondary analysis followed the Strengthening the Reporting of Observational Studies in Epidemiology (STROBE) Statement. See Supplement 1 for the trial protocol and statistical analysis plan.

A total of 33 332 participants without CKD (defined as an estimated glomerular filtration rate [eGFR] ≥60 mL/min/1.73 m^2^) were selected from 33 995 participants in the CRHCP study, and the inclusion and exclusion criteria for the primary study have also been published previously.^9,16,17^ Data on participants' race and ethnicity were collected and are available for reporting. These demographic characteristics were obtained through self-reporting at baseline using a standardized questionnaire. Among participants in the current post hoc analysis, 17 069 participants were randomized to the intervention group and 16 263 to the usual care group. Participants were then stratified by eGFR into 2 groups: 60 to 89 mL/min/1.73 m^2^ (3783 in the intervention group and 3779 in the usual care group) and 90 mL/min/1.73 m^2^ or greater (13 286 in the intervention group and 12 484 in the usual care group) (eFigure 1 in Supplement 2).

### Intervention, Follow-Up, and Measurements

Detailed information regarding the intervention model of the CRHCP has been previously published.^9,16^ In summary, the intervention group received comprehensive care from trained nonphysician community health care professionals with a specific focus on achieving a BP target of 130/80 mm Hg. In the usual care group nonphysician community health care professionals were trained for standard BP measurement but not protocol-based hypertension management. Each participant in the intervention group was given a complimentary home BP monitoring device and monthly supplies of antihypertensive medications at no or reduced cost, along with regular health coaching sessions. Follow-up assessments were conducted every 6 months after enrollment (including demographic information, anthropometric information, adherence test, and BP). Office BP measurements were obtained using an automated BP monitor (Omron HBP-1100U, Omron Corp) after 5 minutes of rest in a seated position for each participant. The mean value of 6 measurements taken during 2 days was used to estimate BP levels. Blood samples were collected at baseline and at 36 months to determine serum creatinine concentration, and eGFR was calculated using the new Chronic Kidney Disease Epidemiology Collaboration creatinine equations.

### Outcomes

To assess the efficacy and safety of the protocol, we followed up participants every 6 months during the 36-month follow-up period and assessed for the occurrence of a composite CVD, including myocardial infarction, stroke, heart failure necessitating hospitalization, or cardiovascular death. The occurrence of composite CVD events was defined as the first occurrence during the follow-up period. An end point adjudication committee unaware of the randomization assignments evaluated cases. In cases of disagreement, a third adjudicator was consulted to reach a consensus.

The primary kidney outcome was defined as an eGFR decrease of 30% or more to a level below 60 mL/min/1.73 m^2^. To assess the potential association of intensive BP control with more severe kidney impairment, we additionally used alternative outcome definitions, including (1) eGFR decrease of 40% to 59 mL/min/1.73 m^2^ and (2) eGFR decrease of 50% to 59 mL/min/1.73 m^2^. Because eGFR was only measured at the 36-month follow-up, kidney outcomes were assessed exclusively at this point. In addition, the safety of the study protocol was measured by adverse effects, including symptomatic and asymptomatic hypotension, injurious falls, syncope, and electrolyte disturbance.

### Statistical Analysis

Continuous variables were reported as means (SDs), whereas categorical variables were reported as numbers (percentages). We conducted a comparison of studies based on village-based randomization using the intention-to-treat principle. The association between BP decrease and eGFR decrease was assessed using generalized linear regression equations. Marginal Cox proportional hazards regression models were used to estimate hazard ratios (HRs) and 95% CIs for cardiovascular events, with a 2-sided *P* < .05 considered statistically significant. In these models, village was treated as a random effect, whereas province, county, and township were considered fixed effects. We examined the duration until follow-up loss or occurrence of the first event without conducting hypothesis testing due to lack of adjustment for multiple comparisons in the reported CIs. Additionally, event rates per 100 person-years were calculated. Modified Poisson regression with cluster-robust variance estimation was used to estimate risk ratios (RRs) and 95% CIs for intervention-related kidney outcomes. Subgroup analyses included adjustments for baseline covariates, such as age, sex, cigarette smoking, use of antihypertensive medication, history of CVD, baseline SBP, low-density lipoprotein cholesterol, and fasting plasma glucose stratification variables. Statistical analysis was conducted with SAS software, version 9.4 (SAS Institute Inc) and R, version 4.2.0 (R Project for Statistical Computing).

## Results

### Baseline Characteristics of the Study Participants

Among the 33 995 patients in the CRHCP, 33 332 patients with a baseline eGFR of 60 mL/min/1.73 m^2^ or less were included in this subgroup analysis (mean [SD] age, 62.8 [9.1] years; 61.3% female and 38.7% male) (eFigure 1 in Supplement 2). The median (IQR) follow-up time was 3.06 (3.03-3.11) years. The baseline characteristics of each group are presented in Table 1. In participants with eGFRs of 60 to 89 mL/min/1.73 m^2^ and those with eGFRs of 90 mL/min/1.73 m^2^ or greater, the intervention group had a younger age, higher SBP, higher diastolic blood pressure, higher body mass index (calculated as weight in kilograms divided by height in meters squared), and more use of antihypertensive medications than the usual care group. In the participants with eGFRs of 60 to 89 mL/min/1.73 m^2^, the baseline mean (SD) eGFR was 80.1 (7.7) mL/min/1.73 m^2^ in the intensive BP control group and 80.2 (7.6) mL/min/1.73 m^2^ in the usual care group, respectively. In patients with eGFRs of 90 mL/min/1.73 m^2^ or greater, the baseline mean (SD) eGFR was 101.6 (6.8) mL/min/1.73 m^2^ in the intensive control group and 101.3 (6.6) mL/min/1.73 m^2^ in the usual care group. In addition, we observed that compared with those with eGFRs of 90 mL/min/1.73 m^2^ or greater, participants with eGFRs of 60 to 89 mL/min/1.73 m^2^ had a significantly higher 10-year risk of atherosclerotic cardiovascular disease.

**Table 1.** Baseline Characteristics of Participants

Characteristic	No. (%) of participants				
	eGFR 60-89 mL/min/1.73 m^2^		eGFR ≥90 mL/min/1.73 m^2^		
	Intervention (n = 3783)	Usual care (n = 3779)	Intervention (n = 13 286)	Usual care (n = 12 484)
Age, mean (SD), y	67.9 (9.0)	68.8 (8.9)	61.1 (8.6)	61.4 (8.4)
Sex				
Female	2399 (63.4)	2367 (62.6)	8012 (60.3)	7644 (61.2)
Male	1384 (36.6)	1412 (37.4)	5274 (39.7)	4840 (38.8)
Educational level				
Primary school or lower	2704 (72.3)	2807 (75.2)	8439 (64.0)	8104 (65.6)
Junior high school	866 (23.1)	748 (20.0)	3864 (29.3)	3493 (28.3)
High school	159 (4.3)	157 (4.2)	777 (5.9)	684 (5.5)
College or higher	12 (0.3)	22 (0.6)	110 (0.8)	77 (0.6)
Cigarette smoking				
Never smoked	2689 (71.9)	2649 (70.9)	9221 (69.9)	8532 (69.1)
Former smokers	358 (9.6)	373 (10.0)	1037 (7.9)	991 (8.0)
Current smokers	692 (18.5)	715 (19.1)	2942 (22.3)	2825 (22.9)
Weekly alcohol drinking	440 (11.8)	475 (12.7)	2331 (17.7)	2184 (17.7)
Physical activity ≥5 times weekly^a^	1562 (41.9)	1661 (44.6)	6795 (51.5)	6452 (52.3)
Duration of hypertension, median (IQR), y	8 (5-13)	8 (5-12)	7 (4-11)	7 (4,10)
Use of antihypertensive medications	2446 (64.7)	2175 (57.6)	7875 (59.3)	6592 (52.8)
History of major cardiovascular disease^b^	957 (25.3)	889 (23.5)	2655 (20.0)	2390 (19.1)
History of diabetes	401 (10.6)	371 (9.8)	1107 (8.3)	1002 (8.0)
BMI, mean (SD)	26.0 (3.9)	25.7 (3.8)	26.0 (3.8)	25.9 (3.8)
Systolic blood pressure, mean (SD), mm Hg	160.1 (19.2)	158.1 (18.1)	156.0 (17.4)	154.5 (16.9)
Diastolic blood pressure, mean (SD), mm Hg	86.9 (11.4)	85.8 (11.2)	88.4 (10.4)	87.8 (10.2)
Total cholesterol, mean (SD), mg/dL	200.9 (40.0)	199.4 (39.4)	193.1 (38.7)	192.7 (38.7)
Low-density lipoprotein cholesterol, mean (SD), mg/dL	111.5 (33.7)	110.1 (32.8)	102.8 (31.1)	102.6 (30.8)
High-density lipoprotein cholesterol, mean (SD), mg/dL	54.4 (13.8)	54.0 (13.6)	56.4 (13.3)	56.2 (13.2)
Plasma glucose, mean (SD), mg/dL	113.9 (40.7)	111.9 (37.8)	110.3 (35.2)	110.4 (35.2)
Uric acid, mean (SD), mg/dL	5.6 (1.4)	5.6 (1.5)	4.9 (1.4)	4.9 (1.4)
eGFR, mean (SD), mL/min/1.73 m^2^^c^	80.1 (7.7)	80.2 (7.6)	101.6 (6.8)	101.3 (6.6)
10-y risk for atherosclerotic cardiovascular disease, mean (SD), %^d^	20.3 (13.6)	20.6 (13.4)	13.0 (10.8)	12.8 (10.4)

Abbreviations: BMI, body mass index (calculated as weight in kilograms divided by height in meters squared); eGFR, estimated glomerular filtration rate.

SI conversion factors: To convert cholesterol, low-density lipoprotein cholesterol, and high-density lipoprotein cholesterol to millimoles per liter, multiply by 0.0259; to convert glucose to millimoles per liter, multiply by 0.0555; to convert uric acid to millimoles per liter, multiply by 0.0595.

^a^ Moderate or heavy physical activity for 30 minutes or more at a time.

^b^ Major cardiovascular disease includes myocardial infarction, stroke, and heart failure requiring hospitalization.

^c^ eGFR was calculated based on the 2021 Chronic Kidney Disease Epidemiology Collaboration creatinine equations.

^d^ Atherosclerotic cardiovascular disease risk was calculated based on the American College of Cardiology/American Heart Association pooled cohort equations.

### Blood Pressure During Follow-Up

**Figure 1.**
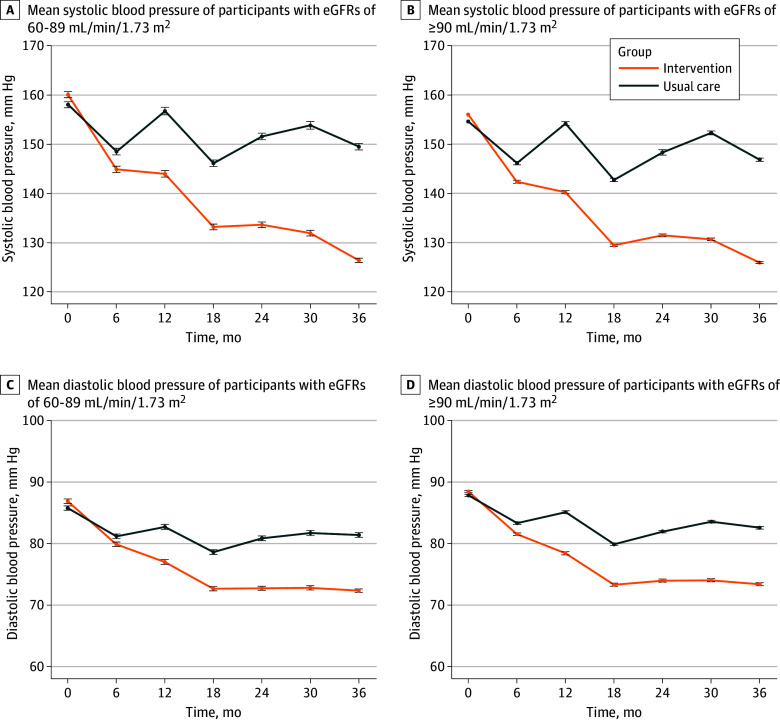
Systolic and Diastolic Blood Pressure During 36 Months of Follow-Up *P* < .001 for interactions between the intervention and follow-up time. Error bars indicate 95% CIs.

Throughout the 36-month follow-up, in participants with eGFRs of 60 to 89 mL/min/1.73 m^2^, the mean (SD) BP of the intervention group was 160.1/86.9 (19.2/11.4) mm Hg at baseline and 126.4/72.3 (13.1/8.4) mm Hg at 36 months; in the same period, the mean (SD) BP of the usual care group was 158.1/85.8 (18.1/11.2) mm Hg at baseline and 149.5/81.4 (19.6/11.1) mm Hg at 36 months (Figure 1). In participants with eGFRs of 90 mL/min/1.73 m^2^ or greater, the mean (SD) BP was 156.0/88.4 (17.4/10.4) mm Hg at baseline and 125.9/73.4 (11.2/7.9) mm Hg at 36 months in intervention group; in the same period, the mean (SD) BP of the usual care group was 154.5/87.8 (16.9/10.3) mm Hg at baseline and 19 146.8 /82.6 (18.0/10.5) mm Hg at 36 months. The use of antihypertensive drugs is detailed in eTable 1 in Supplement 2.

### Cardiovascular Outcomes

**Figure 2.**
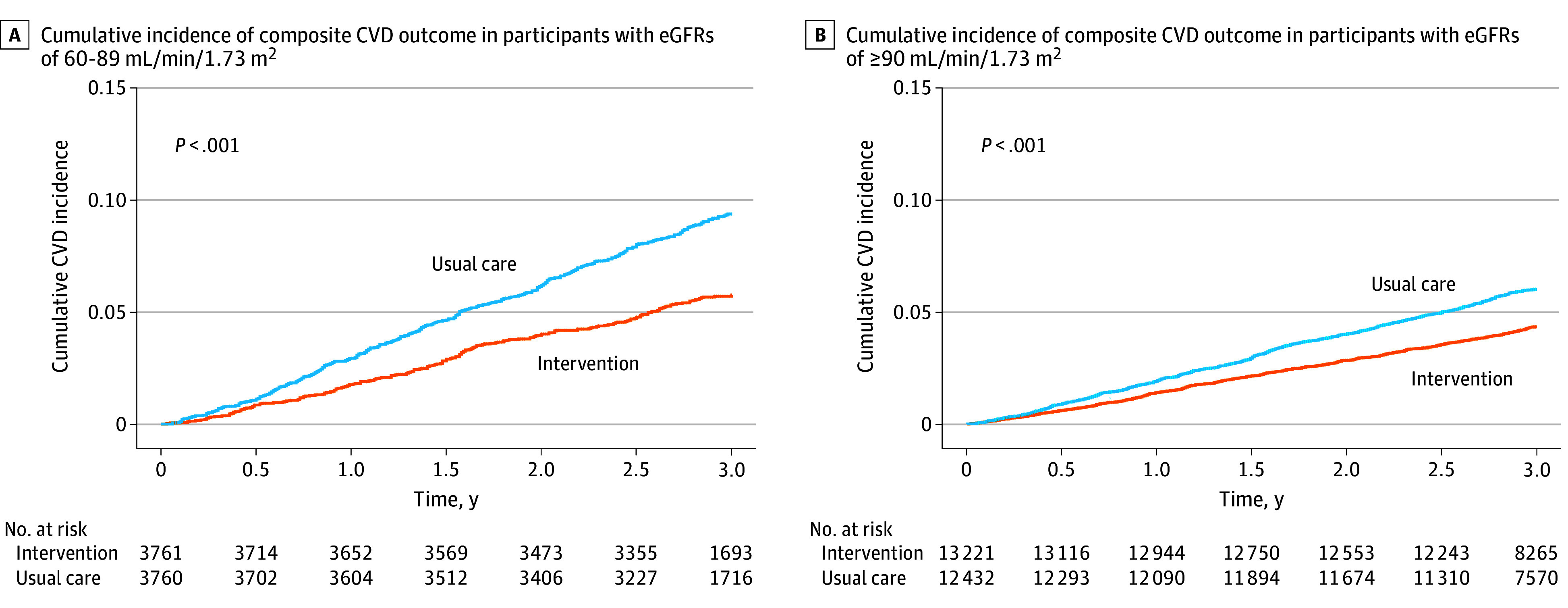
Cumulative Incidence of Composite Cardiovascular Disease (CVD) Outcome in the Intervention vs Usual Care Groups

Among the participants with eGFRs of 60 to 89 mL/min/1.73 m^2^, 210 participants (2.0% per year) in the intervention group and 346 participants (3.3% per year) in the usual care group had the composite CVD outcomes (intervention group hazard ratio [HR], 0.58; 95% CI, 0.49-0.70; *P* < .001); consistent results were obtained after adjustment for baseline covariates (adjusting intervention group HR, 0.59; 95% CI, 0.47-0.68;) (Table 2 and Figure 2). Risk reduction was consistent for the primary outcome and individual components except for death from any cause (Table 2; eFigures 2-6 in Supplement 2). For example, the HRs were 0.60 (95% CI, 0.49-0.73; *P* < .001) for stroke, 0.55 (95% CI, 0.34-0.89; *P* = .02) for myocardial infarction, 0.39 (95% CI, 0.23-0.68; *P* < .001) for heart failure, and 0.69 (95% CI, 0.52-0.93; *P* = .02) for cardiovascular death. However, the HR for all-cause deaths was 0.95 (95% CI, 0.80-1.14; *P* = 0.59).

**Table 2.** Hazard Ratios of Intervention for Cardiovascular Outcomes

Study outcome	Intervention		Usual care		Hazard ratio (95% CI)^a^	*P* value	Adjusted hazard ratio (95% CI)^a^^,^^b^	*P* value	*P* value for interaction
	No. of events	Rate, % per year	No. of events	Rate, % per year					
**CVD **									
eGFR 60-89 mL/min/1.73 m^2^	210	1.98	346	3.32	0.58 (0.49-0.70)	<.001	0.59 (0.47-0.68)	<.001	.11
eGFR ≥90 mL/min/1.73 m^2^	567	1.48	741	2.08	0.70 (0.63-0.78)	<.001	0.67 (0.60-0.75)	<.001	
**Myocardial infarction**									
eGFR 60-89 mL/min/1.73 m^2^	30	0.28	52	0.48	0.57 (0.36-0.92)	.02	0.55 (0.34-0.89)	.02	.09
eGFR ≥90 mL/min/1.73 m^2^	72	0.18	71	0.19	0.94 (0.70-1.26)	.67	0.92 (0.69-1.23)	.58	
**Stroke**									
eGFR 60-89 mL/min/1.73 m^2^	158	1.48	252	2.40	0.61 (0.50-0.74)	<.001	0.60 (0.49-0.73)	<.001	.43
eGFR ≥90 mL/min/1.73 m^2^	459	1.19	624	1.74	0.67 (0.60-0.75)	<.001	0.64 (0.57-0.72)	<.001	
**Heart failure**									
eGFR 60-89 mL/min/1.73 m^2^	16	0.15	36	0.33	0.40 (0.24-0.69)	<.001	0.39 (0.23-0.68)	<.001	.16
eGFR ≥90 mL/min/1.73 m^2^	27	0.07	35	0.10	0.73 (0.46-1.17)	.19	0.72 (0.44-1.16)	.17	
**Death from cardiovascular causes**									
eGFR 60-89 mL/min/1.73 m^2^	65	1.73	91	0.84	0.68 (0.50-0.91)	.009	0.69 (0.52-0.93)	.02	>.99
eGFR ≥90 mL/min/1.73 m^2^	122	0.31	163	0.45	0.69 (0.55-0.87)	.002	0.67 (0.54-0.84)	<.001	
**Death from all causes**									
eGFR 60-89 mL/min/1.73 m^2^	238	2.20	263	2.43	0.92 (0.76-1.11)	.34	0.95 (0.80-1.14)	.59	.36
eGFR ≥90 mL/min/1.73 m^2^	411	1.05	464	1.27	0.83 (0.72-0.95)	.005	0.83 (0.72-0.93)	.003	

Abbreviations: CVD, cardiovascular disease; eGFR, estimated glomerular filtration rate.

^a^ In the Cox proportional hazards regression models, village was used as a random effect.

^b^ Additionally adjusted for age, sex, cigarette smoking, use of antihypertensive medication, history of cardiovascular disease, baseline systolic blood pressure, low-density lipoprotein cholesterol concentration, and fasting plasma glucose concentration.

In participants with eGFRs of 90 mL/min/1.73 m^2^ or greater, the composite CVD outcome appeared in 567 participants (1.5% per year) in the intervention group and 741 participants (2.1% per year) in the usual care group (intervention group hazard ratio [HR], 0.70; 95% CI, 0.63-0.78; *P* < .001); consistent results were obtained after adjustment for baseline covariates (adjusting intervention group HR, 0.67; 95% CI, 0.60-0.75; *P* < .001). The reduction in risk associated with the intervention was also evident for stroke (HR, 0.64; 95% CI, 0.57-0.72; *P* < .001), cardiovascular death (HR, 0.67; 95% CI, 0.54-0.84; *P* < .001), and all-cause death (HR, 0.83; 95% CI, 0.72-0.93; *P* = .003) (Table 2; Figures 2-6 in Supplement 2). Risk reductions for the composite CVD outcome were consistent across subgroups of sex, age, history of CVD, and use of antihypertensive drugs (eFigure 7 in Supplement 2).

### Kidney Outcomes

For kidney outcomes, after 36 months of follow-up, in participants with an eGFR between 60 and 89 mL/min/1.73 m^2^, 121 individuals in the intervention group and 101 in the usual care group experienced a kidney outcome (eGFR decrease of ≥30% to a value <60 mL/min/1.73 m^2^). The RR was calculated as 1.17 (95% CI, 0.84-1.62; *P* = .36), indicating no statistical significance. Similarly, among participants with an eGFR of 90 mL/min/1.73 m^2^ or greater, 107 individuals in the intervention group and 82 in the usual care group had an eGFR decrease of 30% or more to a value less than 60 mL/min/1.73 m^2^, indicating no significant association between the intervention and kidney outcome (RR, 1.21; 95% CI, 085-1.73; *P* = .29). No significant associations were observed when kidney outcome was defined as an eGFR decrease of 40% or more to a value less than 60 mL/min/1.73 m^2^ or an eGFR decrease of 50% or more to a value less than 60 mL/min/1.73 m^2^ (Table 3). In the subgroup analyses of kidney outcomes, no significant statistical heterogeneity was observed in the effect of intensive BP control across different subgroups (eFigure 8-10 in Supplement 2). We also evaluated the association between the decrease in BP and the decrease in eGFR (eTable 2 in Supplement 2). Generalized linear regression analysis demonstrated that for every 1–mm Hg decrease in SBP, the eGFR decreased by 0.023 units (β = 0.023; 95% CI, 0.018-0.029; *P* < .001).

**Table 3.** Risk Ratios of Intervention for Kidney Outcomes

Study outcome	Total events, No. (%)	No. (%) of events		Risk ratio (95% CI)^a^	*P* value	Adjusted risk ratio (95% CI)^a^^,^^b^	*P* value
		Intervention	Usual care				
**eGFR 60-89 mL/min/1.73 m^2^**							
eGFR decrease of ≥30% to <60 mL/min/1.73 m^2^	222 (3.4)	121 (3.7)	101 (3.1)	1.17 (0.84-1.62)	.36	1.14 (0.82-1.58)	.43
eGFR decrease of ≥40% to <60 mL/min/1.73 m^2^	82 (1.3)	49 (1.5)	33 (1.0)	1.46 (0.86-2.49)	.16	1.45 (0.86-2.44)	.17
eGFR decrease of ≥50% to <60 mL/min/1.73 m^2^	41 (0.6)	25 (0.8)	16 (0.5)	1.69 (0.70-4.11)	.25	1.54 (0.58-4.14)	.39
**eGFR ≥90 mL/min/1.73 m^2^**							
eGFR decrease of ≥30% to <60 mL/min/1.73 m^2^	189 (0.8)	107 (0.9)	82 (0.7)	1.21 (0.85-1.73)	.29	1.20 (0.84-1.72)	.31
eGFR decrease of ≥40% to <60 mL/min/1.73 m^2^	152 (0.7)	80 (0.7)	72 (0.6)	1.04 (0.70-1.56)	.83	1.05 (0.70-1.57)	.82
eGFR decrease of ≥50% to <60 mL/min/1.73 m^2^	68 (0.3)	39 (0.3)	29 (0.3)	1.30 (0.71-2.38)	.39	1.27 (0.70-2.32)	.44

Abbreviation: eGFR, estimated glomerular filtration rate.

^a^ Accounted for cluster effects of villages.

^b^ Accounted for cluster effects of villages. Additionally adjusted for age, sex, cigarette smoking, use of antihypertensive medication, history of cardiovascular disease, baseline systolic blood pressure, low-density lipoprotein cholesterol concentration, and fasting plasma glucose concentration.

### Safety Outcomes

We also assessed individuals in each group for the occurrence of safety-related adverse effects (eFigure 11 in Supplement 2). In the group with eGFRs between 60 and 89 mL/min/1.73 m^2^, intensive BP control increased incidence of hypotension (RR, 1.97; 95% CI, 1.26-3.13; *P* = .002) and serum potassium concentration less than 3.0 me/L (to convert to millimoles per liter, multiply by 1) (RR, 4.91; 95% CI, 1.05-46.06; *P* = .02). In the group with eGFRs of 90 mL/min/1.73 m^2^ or greater, the incidence of hypotension (RR, 1.99; 95% CI, 1.58-2.51; *P* = .002) was elevated. Besides that, intensive BP control did not significantly increase the risk of other adverse effects.

## Discussion

We conducted a secondary analysis of CRHCP data to clarify the influence of intensive BP control targeting 130/80 mm Hg on kidney and cardiovascular outcomes in individuals with hypertension without CKD. During a median follow-up period of 3.06 years, our findings demonstrated that intensive BP control was associated with a 42% (95% CI, 30%-51%) reduction of CVD among patients with eGFRs of 60 to 89 mL/min/1.73 m^2^ and a 30% (95% CI, 22%-37%) reduction in patients with eGFRs of 90 mL/min/1.73 m^2^ or greater. Most importantly, intensive BP control was not associated with an increased risk of kidney outcomes. Our subgroup analyses demonstrated consistent associations of intensive blood pressure control with both kidney and cardiovascular outcomes across all subgroups, including age (<60 vs ≥60 years), sex, antihypertensive medication use, history of cardiovascular disease, and history of diabetes.

However, data from the SPRINT study in patients without diabetes and the ACCORD study in patients with diabetes indicated that intensive BP control with the targets of SBP less than 120 mm Hg increased the risk of kidney outcome.^15^ STEP showed that intensive BP control with the SBP target of 110 to 130 mm Hg did not increase the risk of kidney outcome compared with 130 to 150 mm Hg.^8^ The existing evidence remains controversial regarding whether intensive BP control is associated with an elevated risk of kidney outcomes. Our study results demonstrated that intensive BP control did not increase the risk of kidney outcomes, which aligns with the findings from STEP.^8^ On the other hand, all the trials were conducted in selected populations, limiting their generalizability, and our strategy, with a target of 130/80 mm Hg, was based on a general population, with a more representative result. Our research provided supplement evidence about the influence of intensive BP control on kidney function.

Regarding kidney injury, the hydrostatic pressure gradient across the glomerular basement membrane strongly affected glomerular filtration. Therefore, the decrease in SBP would be expected to lower glomerular capillary pressure and subsequently reduce eGFR.^18^ The complexity of hypertension and its interactions with organs made it challenging to establish an ideal BP control target. SPRINT and ACCORD set the intensive target of 120 mm Hg.^10^ Our research set a looser and safer target of 130/80 mm Hg, recommended by AHA guidelines. On the other hand, the rapid decrease of eGFR when applying intensive BP control, mainly occurring in the first 6 months, may be related to the rapid decrease of BP observed in previous studies.^14,19^ The duration of BP lowering in our study lasted for 18 months, which was longer and at a slower rate compared with SPRINT.^10^ This finding might explain why our intensive BP control strategy did not lead to a higher risk of kidney injury.

Generally, our multifaceted BP intervention model was led by the nonphysician community health care professional, which was similar to Control of Blood Pressure and Risk Attenuation–Bangladesh, Pakistan, and Sri Lanka (COBRA-BPS).^20^ However, our study adopted a more stringent BP target (130/80 mm Hg). The results demonstrate that intensive BP control did not increase the risk of kidney outcomes in patients without CKD while providing significant protective effects on cardiovascular outcomes. Specifically, these results had substantial implications for the future hypertension management among the population with mildly impaired renal function. These individuals need to avoid further renal damage and to achieve cardiovascular benefits from lowering BP. This effective, feasible, and sustainable strategy should be integrated into hypertension control programs in low-resource settings in China and worldwide.

### Limitations

There are limitations to our current analysis. First, we relied on eGFR only for the estimate of kidney function but not urinary albumin, which may have led to misclassification of some patients. However, the feasibility of measuring urinary albumin is limited due to the large sample size. Second, the subgroup analysis was not prespecified in the CRHCP study, and the cluster designs resulted in slight imbalances in cluster-level and individual-level baseline covariates. However, we assessed the adjusted HR to control for any imbalances. Third, due to the study design, serum creatinine was only measured at baseline and 36-month follow-up, which might result in a failure to capture temporary kidney function decline. Fourth, we did not exclude cases of acute kidney injury that occurred due to non–BP-related causes, which might have led to potential misclassification of some patients as having BP-related kidney outcomes. Additionally, blood information was obtained only at baseline and 36 months and may not have accurately assessed electrolyte disturbances.

## Conclusions

This secondary analysis of the CRHCP study suggests that intensive BP control with a target of 130/80 mm Hg led by nonphysician community health care practitioners could reduce the incidence of composite CVD without increasing the risk of kidney injury in patients without CKD. These findings provide further evidence supporting the implementation of intensive BP control in patients without CKD.

## Notes

Supplement 1.
Trial Protocol and Statistical Analysis Plan

Supplement 2. **eTable 1.** Types of antihypertensive medications used during the 36-month follow-up among patients. **eTable 2.** Correlation analysis between BP decline and eGFR decline **eFigure 1.** Flow chart of subject inclusion **eFigure 2.** Cumulative incidence of stroke in the intervention versus usual care group **eFigure 3.** Cumulative incidence of myocardial infarction in the intervention versus usual care group **eFigure 4.** Cumulative incidence of heart failure in the intervention versus usual care group **eFigure 5.** Cumulative incidence of cardiovascular death in the intervention versus usual care group **eFigure 6.** Cumulative incidence of all-cause death in the intervention versus usual care group **eFigure 7.** Forest plots of subgroups for the composite cardiovascular outcome **eFigure 8.** Forest plots of subgroups for eGFR decline of ≥30% to a value < 60 ml/min/1.73m^2^ **eFigure 9.** Forest plots of subgroups for eGFR decline of ≥40% to a value < 60 ml/min/1.73m^2^ **eFigure 10.** Forest plots of subgroups for eGFR decline of ≥50% to a value < 60 ml/min/1.73m^2^ **eFigure 11.** Safety outcomes by randomization groups

Supplement 3.
Data Sharing Statement
