# Supplementary material for: Intensive Systolic Blood Pressure Reduction and Kidney and Cardiovascular Outcomes: A Secondary Analysis of a Randomized Clinical Trial
Source: JAMA Netw Open. 2025 Jul 11;8(7):e2519604. doi: 10.1001/jamanetworkopen.2025.19604 (PMC12254891; doi:10.1001/jamanetworkopen.2025.19604)
Supplement: Supplement 1. — Trial Protocol and Statistical Analysis Plan [file jamanetwopen-e2519604-s001.pdf]

China Rural Hypertension Control (CRHC) Project

Study PROTOCOL

March 2018

July 2018

November 2019

January 2020

October 2021

CRHC Project Steering Committee

Shenyang, China

New Orleans, US

This trial is registered with ClinicalTrials.gov, NCT03527719.

This work is supported by the National Key Research and Development Program (Grant #2017YFC1307600), the Ministry of Science and Technology of China

Table of Contents

1. Protocol Summary 3

2. Research Background 6

3. Research Design 7

4. Research Objectives 9

5. Study Participants 9

6. Randomization and Recruitment 10

7. Interventions 10

8. Usual Care 14

9. Data Collection 14

10. Mobile Blood Pressure Management System 15

11. Study Measurements 14

12. Study Outcomes 17

13. Cardiovascular Disease Outcomes 18

14. Dementia and Cognitive Impairment No Dementia 21

15. Statistical Power and Sample Size 244

16. Data Management 255

17. Data Analysis Plan 255

18. Quality Control 277

19. Safety Monitoring 299

20. Study Organization 30

21. Ethical Considerations 33

22. Patient Management during the COVID-19 Outbreak 333

23. Dissemination and Scale-up 333

24. Timeline 344

25. References 366

1. Protocol Summary

The summary of the China Rural Hypertension Control (CRHC) trial is shown in Table 1.

Table 1. The summary of the CRHC trial

**Title**	China Rural Hypertension Control Project
**Background**	Hypertension is the leading preventable risk factor for cardiovascular disease (CVD) and premature death in China. The prevalence of hypertension is high and increasing while the control rate is low, especially in rural areas. Traditionally, village doctors play an important role in infectious disease control and delivering essential health services to rural residents in China.
**Objective**	The overall objective of the CRHC Project is to develop an effective, feasible, and sustainable implementation strategy to achieve more intensive BP control among rural residents in China. Moreover, this implementation trial will test the effectiveness of a lower BP target (<130/80 mmHg) on cardiovascular and cognitive outcomes. Specifically, we will test the effectiveness of a village doctor-led multifaceted intervention, compared with usual care, on BP control, CVD, and all-cause dementia among rural residents with hypertension in China.
**Study Design**	The CRHC Project is a cluster randomized trial that will be conducted in 320 villages from three provinces in mainland China. One hundred and sixty villages will be randomly assigned to a village doctor-led multifaceted intervention and 160 villages to control, stratified by provinces, counties, and townships. A total of 32,000 individuals aged ≥40 years with uncontrolled hypertension will be recruited into the study. The village doctor-led multifaceted intervention is designed to overcome barriers at the healthcare system, provider, patient, and community levels. Study participants are followed every 6 months for BP, CVD, and other study outcomes. The primary outcome is BP control (<130/80 mm Hg) at 18 months in phase 1, CVD events over 36 months in phase 2, and all-cause dementia over 48-month follow-up in phase 3.
**Eligibility Criteria**	<u>Inclusion criteria for study villages:</u><br>• The village has a regular village doctor who is willing to participate in the hypertension control project<br>• The village does not plan to merge with other villages within 3 years<br>• The village is at least 2 kilometers away from other participating villages<br>• The village participates in the China New Rural Cooperative Medical Scheme<br><u>Inclusion criteria of study participants:</u><br>• Men or women aged ≥40 years<br>• Mean untreated systolic BP ≥140 mm Hg and/or diastolic BP ≥90 mm Hg or mean treated systolic BP ≥130 mm Hg and/or diastolic BP ≥80 mm Hg for individuals without a history of clinical CVD; or mean treated/untreated systolic BP ≥130 mm

	Hg and/or diastolic BP ≥80 mm Hg for individuals with a history of clinical coronary heart disease, heart failure, stroke, diabetes, or chronic kidney disease<br>• Have lived in a participating village for at least 6 months<br>• No intention to migrate within next 3 years<br>• Taking part in the New Rural Cooperative Medical Scheme<br>• Not pregnant or planning to become pregnant<br>• No malignant tumors and life expectancy ≥3 years<br>• Willing to participate and able to sign informed consent
**Intervention**	A village doctor-led multifaceted intervention program will be implemented in the intervention group. Village doctors will be trained on standard BP measurement, protocol-based hypertension treatment, and health coaching. They will also receive technical support and supervision from primary care physicians and hypertension specialists and performance-based financial incentives. Study participants will receive health coaching on home BP monitoring, lifestyle changes, and adherence to medications. The village doctor-led multifaceted intervention strategies will target the following barriers at the healthcare system, village doctor, and patient levels.<br>• Health systems<br>− Establishing an organizational structure for chronic disease management<br>− Using hypertension control rate as one of the metrics for resource allocation<br>− Providing the lowest discount or free medications for antihypertensive treatment<br>• Village doctors<br>− Training village doctors to measure BP according to a standard protocol<br>− Training village doctors to use a simple stepwise protocol for BP management<br>− Training village doctors to conduct health coaching on lifestyle changes and medication adherence<br>• Patients<br>− Training patients to self-monitor home BP<br>− Encouraging lifestyle change and medication adherence<br>− Connecting patients for group social support
**Study Outcomes**	Phase 1 will last for 18 months, phase 2 will last for 36 months, and phase 3 will last for 48 months. The primary and secondary study outcomes are as follows:<br><u>Study Outcomes in Phase 1</u><br>Primary outcome: Proportion of hypertension control (BP <130/80 mm Hg) at 18 months<br>Secondary outcome：<br>• Changes in mean systolic and diastolic BP from baseline to 18 months<br>• Proportion of hypertension control (BP <140/90 mmHg) at 18 months<br>• Proportion of patients adhered to antihypertensive medications at 18 months<br><u>Study Outcomes in Phase 2</u><br>Primary outcome: Composite cardiovascular disease (myocardial infarction, stroke,

	heart failure requiring hospitalization or treatment, and cardiovascular death)<br>Secondary outcome:<br>• Individual cardiovascular disease<br>− Myocardial infarction<br>− Stroke<br>− Heart failure requiring hospitalization or treatment<br>− Cardiovascular death<br>• All-cause mortality<br>• Changes in mean systolic and diastolic BP from baseline to 36 months<br>• Aortic dissection<br>• Incident malignant tumors<br><u>Study Outcomes in Phase 3</u><br>Primary outcome: All-cause dementia<br>Secondary outcome:<br>• Cognitive impairment no dementia<br>• Composite outcome of dementia and cognitive impairment no dementia<br>• Death from all causes<br>• Composite outcome of dementia and deaths<br>• Composite and individual cardiovascular disease (myocardial infarction, stroke, heart failure requiring hospitalization or treatment, and cardiovascular death)<br>• Changes in mean systolic and diastolic BP from baseline to 48 months<br>• Proportion of hypertension control (BP <130/80 mm Hg or <140/90 mmHg) at 48 months
**Sample Size**	The sample size of the proposed trial is calculated for the primary outcome of phase 2 due to the minimum required sample size being much larger in phase 2 than in phase 1. The sample size calculation is based on the following assumptions: 160 clusters in each of the intervention and control groups, 2.0% per year CVD event rate in the control group, 25% risk reduction associated with the intervention, average follow-up duration of three years, intra-cluster correlation coefficient (ICC) within villages of 0.025 for CVD events, two-sided significance level of 0.05, and statistical power of 90%. The overall minimum sample size is 32,000 subjects (16,000 in each comparison group and 100 participants in each cluster). For phase 1, we have >99% statistical power to detect a 10% difference in the primary outcome between the two comparison groups assuming 20% of participants in the control and 30% in the intervention group achieve BP <130/80 mmHg at 18 months. An ICC of 0.05 for hypertension control is assumed. The statistical power is calculated using a Z test with a two-sided significance level of 0.05. We also calculated statistical power for phase 3 based on the following assumptions: 163 clusters in each group, 104 participants per cluster, a 5.0% proportion of dementia over 4 years, a 15% risk reduction, an average follow-up duration of 4 years, a lost-to-follow up rate of 1.6% per year, an intra-cluster correlation of 0.001, and a 2-sided significance level of 0.05.

	The overall statistical power was determined to be >85%.
**Statistical Analysis**	Intention-to-treat analyses will be conducted. A 2-sided p-value <0.05 will be considered statistically significant. In phase 1, we will test the difference in the proportions of patients with controlled BP and the difference in mean BP changes between the two comparison groups using a linear mixed-effects model. In phase 2, the cumulative CVD event rates will be calculated using time-to-event methods according to randomization group and stratified by villages. The difference will be tested using a clustered log-rank test with the null hypothesis that cumulative incidences are the same between the two comparison groups. Marginal structural Cox proportional hazards models will be used to assess the effectiveness of village doctor-led intervention vs. control on CVD event rates stratified by village. In a sensitivity analysis, important co-variables such as age, sex, education, duration of hypertension, and unbalanced CVD risk factors between the two comparison groups will be adjusted. In phase 3, the relative risk of dementia and other cognitive impairment outcomes associated with intervention will be calculated using the generalized linear mixed model.

2. Research Background

Hypertension is the top preventable risk factor for CVD and premature death in China, accounting for 50.5% of CVD deaths, 58.8% of stroke deaths, and 21.3% of premature all-cause deaths.[1] It was estimated that 292 million adults in China had hypertension in 2013-14, representing an absolute increase of 139 million individuals from 2002 [2]. When the 2017 American College of Cardiology (ACC)/American Heart Association (AHA) hypertension guideline's definition (≥130/80 mmHg) is applied, the number of adults with hypertension doubles [3]. Although clinical trials have demonstrated that antihypertensive treatment significantly reduces CVD and all-cause mortality, the proportion of controlled hypertension with blood pressure (BP) <140/90 mmHg is extremely low in China, especially in rural areas. In several national surveys, the proportion of controlled hypertension ranged from 7.2% to 15.3% in the general adult population and 4.4% to 13.1% in rural residents.[2-6] Based on the 2017 ACC/AHA guideline, the hypertension control rate (BP<130/80 mmHg) was even lower: 3.0% in the general population and 2.5% in rural residents.[3] The mortalities from CVD and stroke are higher among rural residents than urban residents in China.[7] Uncontrolled hypertension plays an important role in the increased burden of CVD and stroke mortality in rural residents in China.[1,7] Therefore, hypertension control in rural residents has become both a major public health priority and challenge.

Observational epidemiological studies have shown a strong, independent, and linear association between BP and risk of CVD without any evidence of a BP threshold.[8] A meta-analysis of observational studies involving more than one million individuals has indicated that death from both coronary heart disease (CHD) and stroke increases progressively and linearly from levels as low as 115 mmHg systolic

BP and 75 mmHg diastolic BP upward.[8] Randomized clinical trials have demonstrated that BP lowering with commonly used regimens, such as diuretics, angiotensin-converting-enzyme inhibitors (ACEIs), angiotensin-receptor blockers (ARBs), and calcium channel blockers (CCBs), reduces the risk of CVD and all-cause mortality. [9] The Systolic Blood Pressure Intervention Trial showed that intensive BP treatment (systolic BP target of <120 mmHg) was associated with a 25% reduction in CVD events and a 27% reduction in all-cause mortality compared to standard treatment (systolic BP target of <140 mmHg).[10] A network meta-analysis of 42 trials that included 144,220 patients with hypertension reported linear associations between mean achieved systolic BP and risk of CVD and mortality, with the lowest risk among those who achieved a systolic BP of 120-124 mmHg.[11] Therefore, the 2017 ACC/AHA hypertension guideline recommended a BP target of <130/80 mmHg for all adults with hypertension.[12] The 2018 Chinese hypertension guideline, however, recommends a treatment target of <140/90 mmHg for patients aged <65 years and <150/90 mmHg for elderly patients aged ≥65 years.[13,14] The rationale for these less intensive treatment goals includes the lack of data on the benefits of lower BP targets in the Chinese population and poor BP control rates using the current criteria.[13]

Village doctors are a unique group of non-physician health workers with basic medical training who provide basic primary healthcare services in rural China.[15] In recent years, they have been engaging in chronic non-communicable disease management in addition to their traditional roles in infectious disease control, maternal and child health and basic care of common diseases.[16] There were approximately 630 million rural residents in 2013, representing nearly half of China's population. Since these frontline health workers can successfully manage patients with chronic diseases upon receiving training,[16] a village doctor-led intervention could be a sustainable approach to hypertension control in rural China. Accumulating evidence suggest that multilevel and multicomponent strategies were most effective for blood pressure reduction, which targets barriers to hypertension control at health care system, healthcare provider, community, and patient levels.[17-19] However, these multifaceted approaches have not been well tested in randomized trials in rural China.

In the China Rural Hypertension Control (CRHC) Project, a cluster randomized trial, we will evaluate the effectiveness of a village doctor-led multifaceted intervention in improving hypertension control in rural China. We will use a blood pressure target of <130/80 mm Hg as the primary outcome given the recommendation of the 2017 ACC/AHA hypertension guideline and a lack of evidence in China. The primary hypothesis is that the hypertension control rate will be greater in the intervention group compared with that in the usual care group.

3. Research Design

China Rural Hypertension Control Project is a multi-centered cluster randomized controlled trial. We will select a total of 320 villages from Liaoning, Shaanxi, and Hubei provinces. These villages will be

randomly assigned to a village doctor-led multifaceted intervention group (160 villages) and a usual control group (160 villages), stratified by provinces, counties, and townships. All patients with hypertension aged 40 years and older will be recruited into the study. Overall, a total of 32,000 participants will be recruited. The village doctor-led multifaceted intervention is designed to overcome barriers at the healthcare system, provider, patient, and community levels. The intervention program is developed according to our previous experience in rural hypertension management in China as well as proven effective implementation strategies from recent studies.[17] Study participants are followed every six months for BP, CVD, and other study outcomes. The primary outcome is BP control (<130/80 mmHg) at 18 months in phase 1, CVD events over 36 months in phase 2, and dementia and cognitive impairment at 48 months in phase 3 (**Figure 1**).

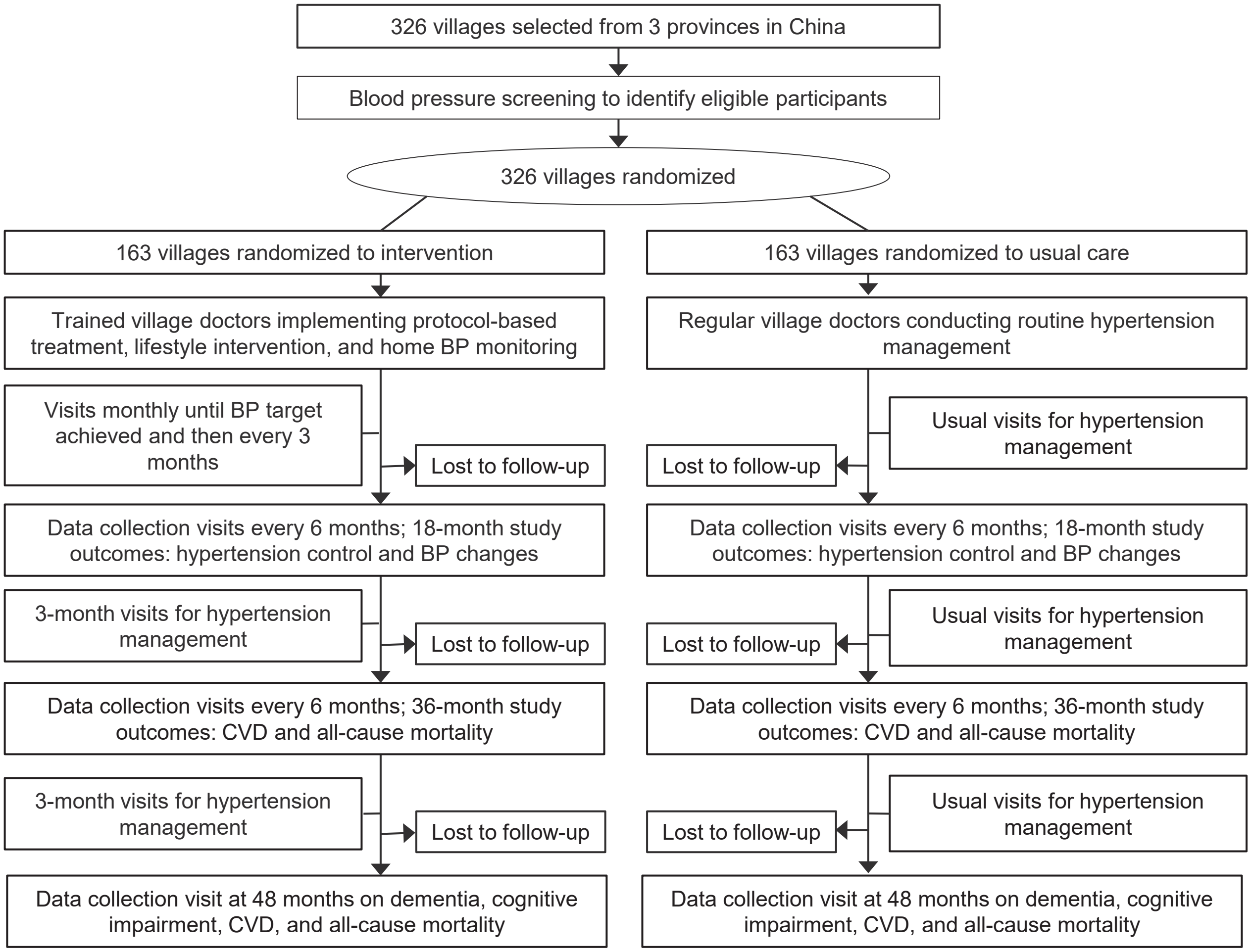

Figure 1. Study Design of the China Rural Hypertension Control Project

4. Research Objectives

The overall objective of the CRHC is to develop an effective, feasible, and sustainable implementation strategy to achieve more intensive BP control in rural residents in China. Moreover, this implementation trial will test the effectiveness of a lower BP target (<130/80 mmHg) on CVD outcomes. The specific aims for phases 1 and 2 of this project are:

Phase 1: to test whether a village doctor-led multifaceted intervention program will improve BP control (< 130/80 mmHg) and reduce BP levels among hypertensive patients over an 18-month period compared to control.

Phase 2: to test whether a village doctor-led multifaceted intervention program will reduce CVD events, as well as mortality from CVD and all causes, among hypertensive patients over a 36-month period compared to control.

Phase 3: to test the effectiveness of a village doctor-led blood pressure intervention program compared to usual care on all-cause dementia and other cognitive impairment outcomes among patients with hypertension over a 48-month period.

5. Study Participants

A total of 320 study villages will be selected. Each village plans to recruit 90-110 participants with hypertension from village-wide BP screenings. In this implementation research, minimal eligibility criteria will be applied to include a representative sample of patients. The eligibility criteria target patients ≥40 years who are at higher risk for CVD.

5.1. Eligibility criteria of study village

- The village has a regular village doctor who is willing to participate in the hypertension control project
- The village does not plan to merge with other villages within 3 years
- The village is at least 2 kilometers away from other participating villages
- The village participates in the China New Rural Cooperative Medical Scheme

5.2. Eligibility criteria of study participants

- Men or women aged ≥40 years
- Mean untreated systolic BP ≥140 mm Hg and/or diastolic BP ≥90 mm Hg or mean treated systolic BP ≥130 mm Hg and/or diastolic BP ≥80 mm Hg for individuals without a history of clinical cardiovascular diseases; or mean treated/untreated systolic BP ≥130 mm Hg and/or diastolic BP ≥80 mm Hg for individuals with a history of clinical coronary heart disease, heart failure, stroke,

diabetes, or chronic kidney disease

- Have lived in a participating village for at least 6 months
- No intention to migrate within next 3 years
- Taking part in the New Rural Cooperative Medical Scheme
- Not pregnant or planning to become pregnant
- No malignant tumors and life expectancy ≥3 years
- Willing to participate and able to sign informed consent

6. Randomization and Recruitment

The randomization unit will be villages, stratified by provinces, counties, and townships. The randomization schedules will be generated using SAS programming with 1:1 allocation ratio. The randomization process will be conducted at Tulane University Translational Science Institute in the US.

We aim to recruit all eligible patients with hypertension in each village, which may vary from 80-120. Village doctors will generate a list of hypertensive patients in each village. Patients will be informed by village doctors about this trial. After signing an informed consent, the participants will be assessed for eligibility by the research team. The investigator team will determine eligibility of the participants based on the inclusion and exclusion criteria. Those participants who meet the criteria will be enrolled into the trial.

7. Interventions

A simple stepped-care protocol for BP treatment adapted from the 2017 ACC/AHA hypertension guideline and the 2018 Chinese hypertension guideline will be used (**Figure 2**).[12,13] Initiation of treatment is based on clinic BP (mean of six BP readings from two visits) ≥140/90 mmHg among individuals without a history of clinical CVD and ≥130/80 mmHg among individuals with a history of clinical CVD. Antihypertensive medication adjustments are based on clinic BP at a single visit (mean of three readings) for a target systolic BP <130 mmHg and diastolic BP <80 mmHg. Based on participant BP levels, monotherapy (CCB, ACEI, ARB, or thiazide-type diuretic) or combined therapy (CCB plus diuretic, CCB plus ACEI or ARB, or diuretic plus ACEI or ARB) will be used unless comorbidities require specific agents.

The village doctor-led multifaceted intervention strategies will target the following barriers at the healthcare system, village doctor, and patient levels.[18,19]

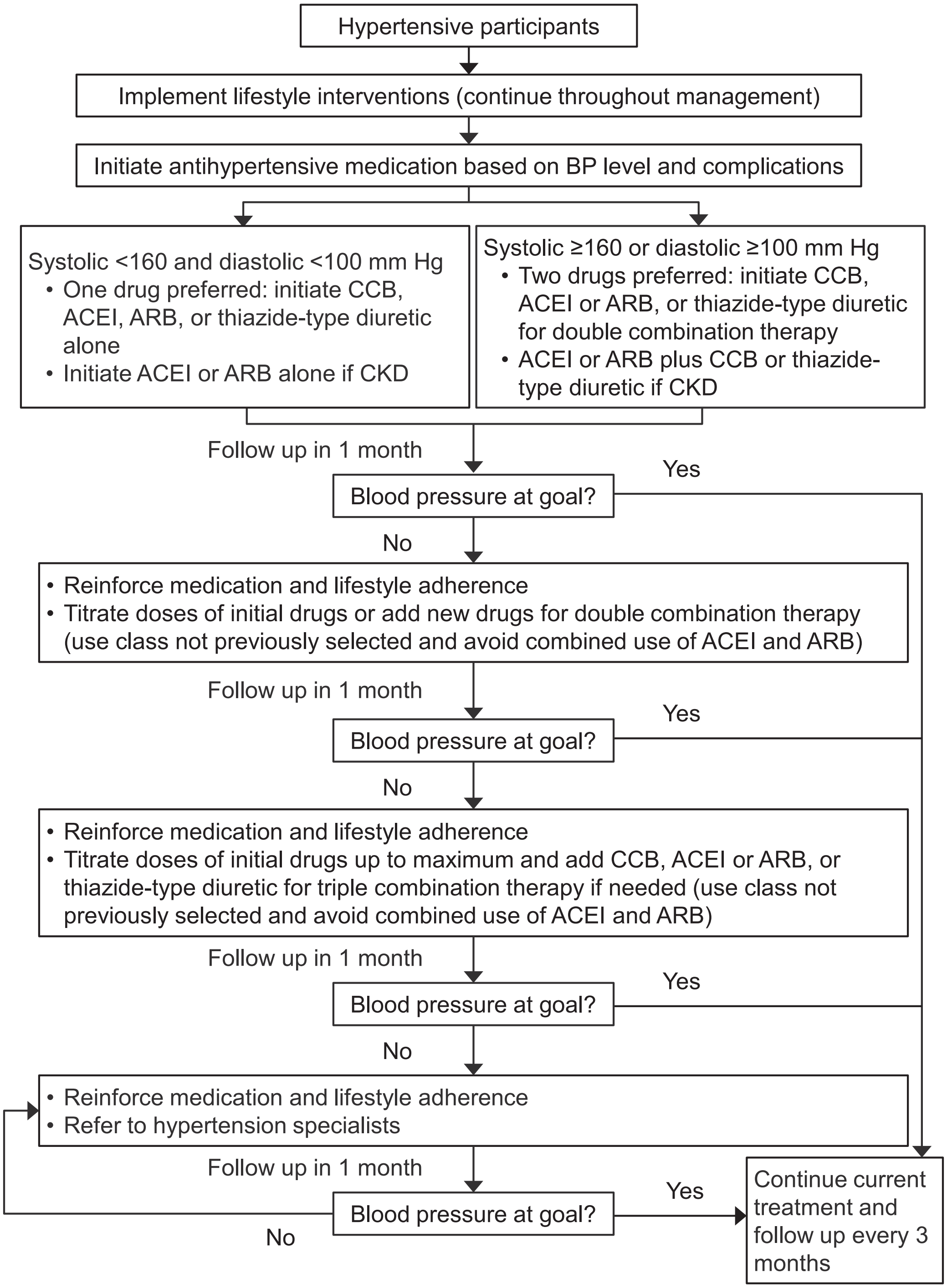

Figure 2. Treatment protocol of the China Rural Hypertension Control Project. Abbreviations: ACEI, angiotensin-converting enzyme inhibitor; ARB, angiotensin receptor blocker; BP, blood pressure; CCB, calcium channel blocker; CKD, chronic kidney disease.

7.1. Interventions on health system

7.1.1. Establishing an organizational structure for chronic disease management

A hierarchical organizational structure for chronic disease management at the city or county, township, and village levels will be established in each participating city and county. Village doctors are responsible for routine hypertension management, including standardized BP measurement, initiation or titration of antihypertensive medications, health coaching on lifestyle modification and medication adherence, and patient follow-up visits. A hypertension management monitoring team will be established, which includes primary care physicians from township hospitals or centers for disease control who meet with village doctors regularly. The primary care physicians will instruct village doctors on individual patient management and compliance to medication to best achieve the BP targets. Village doctors are supported and supervised by the team, who periodically evaluate the performance of village doctors on hypertension management. Hypertension specialists at city or county hospitals are responsible for training village doctors and consulting with them on hypertension management. The specialists provide guidance to village doctors on patients who require >3 antihypertensive medications. They also manage patients with complications or resistant hypertension referred by village doctors. Hypertension specialists communicate with village doctors monthly through meeting or internet.

7.1.2. Using hypertension control rate as one of the metrics for resource allocation

A reward system for village doctors' performance on hypertension management will be established. The performance indicators include number and frequency of BP measurement, number and frequency of lifestyle intervention sessions, intensification of antihypertensive medications based on treatment protocol, participants' adherence to medication, BP control rate, and patient follow-up rate. Hypertension control will be the most important indicator for management performance assessment and determinant for financial rewards to village doctors.

7.1.3. Providing the lowest discount or free antihypertensive medications

We aim to provide affordable and effective antihypertensive medications to all patients in the intervention villages. We will provide the lowest discount antihypertensive medications to all patients in the intervention villages according to the reform plan of the China New Rural Cooperative Medical Scheme, which aims to increase health insurance reimbursement and reduce medication costs for all patients. In addition, free antihypertensive medications (including nitrendipine tablets, captopril tablets, indapamide tablets, nifedipine controlled release tablets, amlodipine besylate tablets, enalapril maleate tablets, valsartan capsules and lisinopril hydrochlorothiazide), which will be purchased by the CRHC or will be donated by pharmaceutical companies, will be provided to low-income patients or those who require additional assistance as determined by village doctors. Through these strategies, the patients' access and compliance to antihypertensive medication will be improved.

7.2. Intervention on village doctors

7.2.1. Training village doctors to measure BP according to a standard protocol

Village doctors in the intervention group will be trained in standardized BP measurement methods. Training materials, including videos, will be sent to each village doctor before on-site training. The standardized BP measurement method is based on the 2017 ACC/AHA hypertension guideline and the 2018 Chinese hypertension guideline [12,13]. A certification process including testing for knowledge and BP measuring skills will be given after a one-day on-site training.

7.2.2. Training village doctors to use a simple stepwise protocol for BP management

A stepwise protocol for hypertension management, including treatment algorithm, medication selection, contraindications of medications, and adjustment strategies, will be provided to village doctors. Village doctors will receive a series of trainings in the first six months. Tests, including related questions and simulated patient scenarios, will be given to ensure the village doctors will manage their patients according to the protocol.

7.2.3. Training village doctors to conduct health coaching on lifestyle changes and medication adherence

Village doctors will also be trained in health coaching on lifestyle modifications (e.g., reducing dietary sodium intake and alcohol consumption), medication side effects, and medication adherence, as well as tracking and managing patients using the internet and mobile devices.

7.3. Interventions on patients

7.3.1. Home blood pressure monitoring

Study participants in the intervention group will receive an electronic BP monitor and will be trained to measure their BP 2-3 days a week and record the readings in a BP log. The electronic BP monitor will promote the habit of self-monitoring home BP among the patients. The patients will be encouraged to communicate with the village doctors actively to enhance their BP self-management consciousness.

7.3.2. Lifestyle change and medication adherence

Study participants will receive health coaching from village doctors and health education materials covering various topics such as BP and risk of CVD, BP treatment targets, side effects of antihypertensive medications, and lifestyle modifications. Health coaching will be conducted at individual patient visits or at group sessions either in-person or through mobile devices. A BP self-management reward system will be established. Patients will accumulate reward points according to their performance on self-blood pressure measurements, lifestyle change, medication adherence, follow-up visits, and health coach session participation. Based on quarterly and annual reward points, patients will receive gifts to encourage self-management of BP, especially lifestyle change and medication adherence.

7.3.3. Social support

Social support groups among participants and family members will be established in each intervention village. Patients will take part in group activities periodically to discuss their experiences with BP measurements, lifestyle change, and medication adherence. They will also give feedback to village doctors. They can meet in-person or by social media (i.e., WeChat) and telephone.

8. Usual Care

Village doctors who are randomly assigned to the control group will not receive intervention training or support. However, they will be trained in standardized BP measurement. By the end of this study, they will receive training on the protocol-based management strategies if this approach is proven effective in the proposed trial.

9. Data Collection

The study data will be collected at screening and baseline visits as well as at follow-up visits every 6 months by trained study staff (**Table 2**). A questionnaire on demographic information, lifestyle factors, medical history and adherence to medication will be administrated by the study staff at the screening visit and follow-up visits. Three BP measurements will be obtained at each visit by trained, certified observers according to a standard protocol recommended by the AHA.[20] Body height and weight will be measured with patients in light clothing without shoes according to a standard protocol. Two measurements will be obtained at each visit, and their mean values will be used for data analyses. A 12-hour fasting blood sample will be collected in the morning from participants at baseline and 36-month follow-up visits to measure glucose, lipids, electrolytes, liver and renal function, and other routine blood biochemical indexes in a certified central clinical laboratory.

Table 2. Study visit and data collection schedule

Data Collection	SV	BL	Follow-Up Visits, Months						
			6	12	18	24	30	36	48
Informed consent	X								
Questionnaire	X		X	X	X	X	X	X	X
Blood pressure	X	X	X	X	X	X	X	X	X
Weight/height	X		X	X	X	X	X	X	X
Medication adherence			X	X	X	X	X	X	X
Biochemistry profile		X						X	
CVD outcome assessment			X	X	X	X	X	X	X
Dementia/cognitive impairment outcomes									X

SV = screening visit; BL = baseline visit

CVD outcomes will be assessed at every 6-month follow-up visit. If a study outcome is reported, hospital charts will be requested. All hospital charts will be reviewed by an Outcome Assessment Committee that is blinded to study intervention. Vital information will be updated at each follow-up visit. If a death is reported, the death certificate will be obtained.

At the 48-month follow-up visit, trained and certified neurologists who are blinded to randomization assignment will collect data on medical history, neurological examination, and cognitive function. The Mini–Mental State Examination questionnaire (MMSE) will be administered to all participants in person, while the Functional Activities Questionnaire (FAQ) and Quick Dementia Rating System (QDRS) scales will be administered to knowledgeable informants (family members, village doctors, or both together). In addition, a detailed medical history and a clinical neurological examination will be conducted. All-cause dementia and cognitive impairment no dementia will be diagnosed according to standard criteria by a panel of neurologists who are blinded to the randomization assignment.

10. Mobile Blood Pressure Management System

The CRHC Project will use a mobile BP management system (Shenyang DongRuanXikang Medical System Co., Ltd, Shenyang, China). This system includes an automatic BP monitor (Omron HBP-1100, Tokyo, Japan) and an electronic scanner which connect to a wireless network. Individuals' resident identity cards (an official identity document for personal identification in China) will be scanned prior to BP measurements to confirm participants examined. The devices can store BP readings and upload them to a network platform at the study data coordinating center. Project staff and village doctors will be trained to use this system. Village doctors, primary care physicians, hypertension specialists, and research team members have access to this system and real-time BP data.

11. Study Measurements

11.1. Questionnaire

A questionnaire will be administered in-person by trained and certified research staff. The questionnaire will include sociodemographic information (e.g., age, sex, education, duration of current residency, and health insurance), medical history (e.g., hypertension, diabetes, coronary heart disease, stroke, heart failure, chronic kidney disease, and cancer), lifestyle factors (e.g., smoking, alcohol drinking, physical activity, and sodium and fruit and vegetable consumption), and medication use and adherence.

11.2. Blood pressure measurement

11.2.1. Preparation before blood pressure measurement

Before BP measurement, participants will be informed in detail about the measurement process and their questions will be answered. BP will be measured in a separate, quiet room, with temperature around 25°C. Participants will be advised to avoid alcohol, cigarettes, coffee/tea and exercise for at least 30 minutes

before their BP measurement. BP will be measured with the participant in a seated position after 5 minutes of quiet rest. The patient must be quiet and silent during the BP measurement. At the end of the study, they will be notified of their BP results.

11.2.2. Blood pressure monitor

BP will be measured using an auto-BP monitor (Omron HBP-1100, Tokyo, Japan), which is connected to an ID scanner developed by the project. Patient information is checked before BP measurement and the results are uploaded automatically after measurement. Auto BP monitors will be calibrated before use and every 6 months thereafter.

11.2.3. Blood pressure cuff

A suitable cuff is necessary for accurate BP measurement. The project will provide a variety of cuffs in different sizes for BP measurement. The cuff should have a bladder length that is 80% and a width that is at least 40% of arm circumference. The recommended cuff size is shown below: [20]

- Upper arm circumference 22-26 cm, cuff size 12 cm×22 cm (adult small)
- Upper arm circumference 27-34 cm, cuff size 16 cm×30 cm (adult standard)
- Upper arm circumference 35-44 cm, cuff size 16 cm×36 cm (adult large)
- Upper arm circumference 45-52 cm, cuff size 16 cm×42 cm (thigh).

11.2.4. Measurement procedure

After electronic scanning of ID cards, study participants will be instructed to sit in a backrest seat with their feet flat on the ground and their bare upper arm supported at the level of their heart. Select an appropriate-sized cuff and tie the cuff tightly to the upper arm of the participant. The lower end of the cuff should be 2-3 cm above the antecubital fossa. Keep quiet during the measurement. After the measurement is completed, the record will be automatically uploaded to the network platform for storage by clicking the upload button on the device. The measurement should be repeated at an interval of 1 min for 3 times, and its mean value will be used for analysis.

11.3. Anthropometric measures

During each visit, trained staff will take anthropometric measurements on individuals in light clothing without shoes using a standard protocol.

11.3.1. Body weight

Body weight will be measured to the nearest 0.1 kg on a dedicated scale that will be calibrated weekly. Two repeat measurements will be obtained, and their mean will be used for analyses.

11.3.2. Body height

Body height will be measured to the nearest 0.1 cm with a free-standing stadiometer and the mean value of 2 measurements will be used in analyses.

11.3.3. Waist circumference

Waist circumference will be measured (at the midpoint between the lower margin of the last palpable rib and the top of iliac crest) in centimeters to the nearest 0.1 cm. Two measurements will be obtained, and the mean value will be used for analyses.

11.4. Laboratory tests

Blood samples after a 12-hour overnight fasting will be collected at baseline and at 36-month visits to measure biochemical parameters. Glucose, lipids, electrolytes, liver and renal function, and other routine blood biochemical indexes will be measured in a certified central clinical laboratory. Laboratory results will be used to determine and adjust treatment of hypertension. In addition, serum and plasma are stored for future analysis of other novel cardiovascular risk factors.

12. Study Outcomes

The phase 1 primary outcome is the proportion of participants with BP <130/80 mmHg at 18 months. The secondary outcomes include mean changes in systolic and diastolic from baseline to 18 months, the proportion of participants with BP <140/90 mmHg, and the proportions of participants treated with and adherent to antihypertensive medications. The phase 2 primary outcome is composite CVD (myocardial infarction, stroke, heart failure requiring hospitalization or treatment, and CVD death) over 36 months. The secondary outcomes include individual CVD, all-cause mortality, and mean BP changes from baseline to 36 months. The phase 3 primary outcome is all-cause dementia over 48 months. The secondary outcomes include cognitive impairment no dementia, composite outcome of dementia and cognitive impairment no dementia, CVD, all-cause mortality, BP changes from baseline to 48 months.

12.1. Study outcomes in phase 1

Primary outcome: Proportion of hypertension control (BP <130/80 mm Hg) at 18 months

Secondary outcome:

- Changes in mean systolic and diastolic BP from baseline to 18 months
- Proportion of hypertension control (BP <140/90 mmHg) at 18 months
- Proportion of patients adherent to antihypertensive medications at 18 months

12.2. Study outcomes in phase 2

Primary outcome: Composite cardiovascular disease (myocardial infarction, stroke, heart failure requiring

hospitalization or treatment, and cardiovascular death)

Secondary outcome:

- Individual cardiovascular disease
  - Myocardial infarction
  - Stroke
  - Heart failure requiring hospitalization or treatment
  - Cardiovascular death
- All-cause mortality
- Changes in mean systolic and diastolic BP from baseline to 36 months

12.3. 3. Study outcomes in phase 3

Primary outcome: All-cause dementia

Secondary outcome:

- Cognitive impairment no dementia
- Composite outcome of dementia and cognitive impairment no dementia
- Death from all causes
- Composite outcome of dementia and deaths
- Composite and individual cardiovascular disease (myocardial infarction, stroke, heart failure requiring hospitalization or treatment, and cardiovascular death)
- Changes in mean systolic and diastolic BP from baseline to 48 months
- Proportion of hypertension control (BP <130/80 mm Hg or <140/90 mmHg) at 48 months

13. Cardiovascular Disease Outcomes

13.1. Definition of CVD study outcomes

Myocardial infarction (MI): MI will be defined as the death of part of the myocardium due to an occlusion of a coronary artery from any cause, including spasm, embolus, thrombus, or rupture of a plaque. We will use standard case definitions for both fatal and nonfatal MI based on the combination of symptoms, elevation in biomarkers, and/or ECG findings.[21] The study adjudicators will be guided by pre-specified definitions and operational rules. MI will be identified as detection of a rise and/or fall of cardiac biomarker values (preferably cardiac troponin) with at least one value above the 99th percentile upper reference limit and with at least one of the following:[21]

- Symptoms of ischemia.
- New or presumed new significant ST-segment-T wave (ST-T) changes or new left bundle branch block (LBBB).
- Development of pathological Q waves in the ECG.
- Imaging evidence of new loss of viable myocardium or new regional wall motion abnormality.
- Identification of an intracoronary thrombus by angiography or autopsy.

Stroke: We will use standard case definitions for both fatal and nonfatal stroke. Stroke will be defined based on all available data, including symptoms and signs, and imaging of the brain and large vessels. Adjudicators will use their clinical judgment based on the available evidence to classify each case and will be guided by pre-specified definitions and operational rules. Stroke is generally defined as neurological deficit of cerebrovascular cause that persists beyond 24 hours or is interrupted by death within 24 hours.[22] Exclusionary conditions for stroke include major brain trauma, intracranial neoplasm, coma due to metabolic disorders or disorders of fluid or electrolyte balance, peripheral neuropathy, or central nervous system infections. Stroke will be classified as ischemic stroke, subarachnoid hemorrhage, intraparenchymal hemorrhage, other hemorrhage, other type, or unknown type. In the proposed trial, ischemic stroke is defined as a new lesion detected by computed tomography or magnetic resonance imaging or, in the absence of a new lesion on available imaging, clinical findings consistent with the occurrence of stroke that lasted for more than 24 hours.[23,24]

Heart failure (HF) requiring hospitalization or treatment: HF will be defined as hospitalization, or emergency department visit requiring treatment with infusion therapy, for a clinical syndrome that presents with multiple signs and symptoms consistent with cardiac decompensation/ inadequate cardiac pump function. Only a hospitalization for decompensated HF or an emergency department visit where decompensated HF was diagnosed and intravenous treatment was given will be a potential HF outcome. Thus, diagnosis and treatment of HF by a physician or other provider in the office or clinic setting without hospital admission or an emergency department visit without intravenous therapy for decompensation will not be considered a HF outcome. Adjudication will use the ARIC study adjudication system.[25,26] HF outcome will include definite or possible acute decompensation, including HF with preserved left ventricular ejection fraction as well as HF with reduced ejection fraction. HF will include a variety of clinical presentations, including acute or subacute HF as the primary reason for hospital admission or for emergency department visit where HF was diagnosed, and intravenous treatment was given. The identification and classification of HF cases will rely on multiple pieces of key clinical data as well as adjudicators' clinical judgment, guided by specific, pre-specified definitions and operational rules. No identification of HF should rely on a single piece of data such as the presence of dyspnea or of edema, a low ejection fraction, or an increased brain natriuretic peptide (BNP) value. Adjudicators will use both the

available data and clinical judgment to distinguish between "definite" and "possible" decompensated HF. "Definite" decompensated HF will be assigned when decompensation is clearly present based on available data (satisfies criteria for decompensation). "Possible" decompensation will be assigned when decompensation is possibly but not definitively present, typically where the presence of co-morbidity could account for the acute symptoms (COPD exacerbation, for example).

Cardiovascular death: We will use standard case definitions for classification of CVD death. Definite CVD events will be defined based on temporal relationship to a documented event (e.g., hospitalization for MI, stroke, or HF), or postmortem findings of an acute CVD event. Stroke deaths will be categorized based on the temporal relationship between the stroke event and death in cases where the underlying cause of death is attributed to stroke. Proximal stroke death is a death attributed to stroke and occurring within 30 days of stroke; remote stroke death is underlying cause attributed to stroke and more than 30 days from stroke to death. Probable coronary heart disease (CHD) death will be defined based on findings consistent with chronic CHD, prior history of CHD or documented symptoms consistent with CHD prior to death, and the absence of another likely cause of death. Possible fatal CHD will be adjudicated based on death certificate information consistent with an underlying CHD cause and no evidence of a non-coronary cause. Deaths due to HF will also be adjudicated as CVD deaths.

Aortic dissection: The diagnosis is based on clinical symptoms, signs, and confirmed by typical findings on computed tomography or echocardiography, or surgical and endovascular procedures [27].

Incident malignant tumors: All incident malignant tumor cases will be confirmed by medical records from county, city, or higher-level hospitals.

13.2. CVD outcome adjudication process

Potential clinical outcome events will be identified through 6-month follow-up visits and additional reports from village doctors during the follow-up period. Once the potential outcome reports are received, the study staff at the data coordinating center will request medical records, including hospital discharge summary, laboratory tests, and imaging data evidence from field research teams. All deaths should be verified by relevant medical records and death certificates. All collected medical records, laboratory tests, imaging data, and death certificates will be sent to the Outcome Adjudication Committee members for evaluation. Each case will be independently adjudicated by two committee members to determine if the case meets the diagnosis criteria established for the CRHC study. If there is disagreement on the diagnosis, a third reviewer will be included in the discussion to reach a consensus. The randomization assignment will be blinded to the Outcome Adjudication Committee members. The documents collected for each case will be archived and stored securely.

14. Dementia and Cognitive Impairment No Dementia

14.1. Ascertainment of cognitive status

At the 48-month follow-up visit, trained and certified neurologists who are blinded to randomization assignment will collect data on medical history and risk factors for dementia (e.g., history of stroke, traumatic brain injury, Parkinson's disease, epilepsy, vitamin B12 deficiency, thyroid disease, atrial fibrillation, brain tumor, hyposomnia/insomnia, other sleep disorders, and mental illness, as well as history of carbon monoxide poisoning and pesticide poisoning, and use of medications). They will also conduct a neurological examination (including an assessment of motor and sensory skills, balance and coordination, muscle tone and strength, mental status, reflexes, and functioning of the nerves).

The trained and certified neurologist will administer the Mini-Mental State Examination (MMSE) to study participants in person. The MMSE is a widely recognized 30-point questionnaire extensively employed in clinical and research settings to screen for cognitive impairment.[28] It assesses various cognitive domains, including orientation to time and place (10 points), registration (3 points), attention and calculation (5 points), recall (3 points), as well as language skills and visuospatial abilities (9 points). Importantly, the MMSE has been translated into and culturally adapted to Chinese and validated in the Chinese population.[29,30]

Regardless of the participants' MMSE score, the Functional Activities Questionnaire (FAQ) and Quick Dementia Rating System (QDRS) scales will be administered to knowledgeable informants (family members, village doctors, or both together).

The FAQ assesses instrumental activities of daily living (IADLs), which include tasks such as preparing balanced meals and managing personal finances. This questionnaire demonstrates good internal consistency and exhibits high discriminative validity for differentiating functional independence among patients with dementia and mild cognitive impairment (MCI) due to Alzheimer's disease (AD). It also shows high predictive validity in identifying those at risk of progressing from MCI to AD or from normal aging to MCI. [32]

The FAQ is a brief inventory consisting of 10 measures assessing difficulties in instrumental activities of daily living, namely:

1. Managing simple finances (e.g., paying bills)
2. Handling complex finances (e.g., conducting business affairs)
3. Shopping (e.g., buying groceries)
4. Pursuing hobbies (e.g., playing games)
5. Simple cooking (e.g., heating water)

6. Complex cooking (e.g., preparing a balanced meal)
7. Staying informed about current events
8. Understanding TV, books, or magazines
9. Remembering appointments (e.g., family occasions, holidays, and medication schedules)
10. Maintaining mobility (e.g., driving or traveling out of town)

Each item is rated on a 0-3 scale, where 0 indicates normal, 1 suggests difficulty but self-sufficiency, 2 implies the need for assistance, and 3 signifies dependency. The participant's overall FAQ score is calculated by summing the scores for each of the 10 activities. A cut-off score of 9 (indicating dependence in three or more activities) is recommended to identify impaired function and potential cognitive impairment. Furthermore, the FAQ has been translated into and culturally adapted to Chinese and validated in the Chinese population.[33]

The QDRS, a rapid dementia staging tool, will be completed by an informant (spouses, adult children, and/or village doctors).[34] The QDRS offers a brief yet valid and reliable assessment of the presence and severity of dementia. QDRS scores range from 0 to 30, with higher scores indicating greater cognitive impairment. It encompasses ten domains, including:

1. Memory and recall
2. Orientation
3. Decision-making and problem-solving abilities
4. Activities outside the home
5. Function at home and hobbies
6. Toileting and personal hygiene
7. Behavior and personality changes
8. Language and communication abilities
9. Mood
10. Attention and concentration

These domains effectively capture prominent symptoms of cognitive impairment and dementia. Each domain has five possible answers that characterize changes in the patient’s cognitive and functional abilities. The informant is asked to compare the patient's current state with how they used to be; the key feature is change, and no specific timeframe for change is required. The informant should select the answer that best describes the patient in each category. The QDRS has been validated in various

populations.[34,35] It has also been translated into Chinese for use with Chinese-speaking participants in the MESA-MIND study.

Training in cognitive function assessments will include two stages. In the first stage, key investigators (neurologists) will be trained and certified by Dr. Clara Li, a clinical neuropsychologist at Icahn School of Medicine at Mount Sinai in New York, NY. She has extensive experience in training doctors who are bilingual in Chinese and English to administer neuropsychological tests and interpret cognitive data. In the second stage, all data collectors will be trained and certified to administer cognitive tests by certified investigators.

14.2. Definition of dementia and cognitive impairment no dementia

The primary outcome is all-cause dementia, and the secondary outcomes include cognitive impairment no dementia (CIND), a composite outcome of dementia and CIND, and a composite outcome of dementia and deaths. Mortality will be included as a secondary outcome because cognitive function data are not available for participants who deceased during follow-up.

14.2a. All-cause dementia

The diagnostic criteria for all-cause dementia have been adopted from the Recommendations of the National Institute on Aging-Alzheimer's Association workgroups on diagnostic guidelines for Alzheimer's disease.[36] Specifically, Specifically, a diagnosis of dementia requires the simultaneous presence of following four conditions by integrating data from the MMSE, QDRS, FAQ, medical and psychiatric history, and clinical neurology examination:

1. An expert adjudication panel confirms the presence of significant cognitive impairment.
2. Cognitive or neuropsychiatric symptoms interfere with the ability to function at work or in usual activities.
3. Cognitive and physical function represent a decline from previous levels of functioning and performance.
4. Cognitive impairments are not explained by delirium or major psychiatric disorders.

MMSE score below the normal value for the corresponding education level: MMSE $\leq$23 for middle school and above (7 or more years of education), MMSE $\leq$19 for elementary school (1-6 years of education) and MMSE $\leq$16 for illiterate will be used as one of many indicators for cognitive impairment.[30] No attempt to classify dementia subtype will be made.

14.2b. Cognitive impairment no dementia (CIND)

The diagnostic criteria for CIND have been adopted from the Recommendations of the National Institute on Aging-Alzheimer's Association workgroups on diagnostic guidelines for Alzheimer's disease.[37] In this study, the diagnosis of CIND requires meeting the following four conditions:

1. Confirmation of cognitive impairment by an expert adjudication panel.
2. Evidence of concern regarding a decline in cognition from previous levels.
3. Preserved functionality and independence at work or in usual activities
4. Not being demented

14.2c. Composite outcome of dementia and CIND

Composite outcome of dementia and CIND includes dementia or cognitive impairment no dementia.

14.3. Adjudication of dementia and cognitive impairment no dementia

The final diagnosis of all-cause dementia or CIND will be determined by an expert adjudication panel that is blinded to the intervention assignment. Study records, including medical and psychiatric history, neurological examination findings, cognitive and functional assessments, will be used to adjudicate cognitive status. Participants will be categorized into one of three primary groups: no cognitive impairment, CIND, or dementia. Unclassifiable cases will be placed in a 'cannot classify' category and treated as missing data in all analyses.

Each case will be independently reviewed by two adjudicators using standardized diagnostic criteria. If the two reviewers agree on the diagnosis, it will be recorded as the final diagnosis. If the two reviewers disagree, a third and more experienced adjudicator will join the review and discussion. If a consensus cannot be reached through this additional process, the disagreements will be discussed by the entire panel during regularly scheduled meetings. The classification decision is made by a majority vote of the panel members. There will be no attempt to classify dementia subtypes.

15. Statistical Power and Sample Size

The sample size of the proposed trial is calculated for the primary outcome of phase 2 due to the minimum required sample size being much larger in phase 2 than phase 1. The sample size calculation is based on the following assumptions: 160 clusters in each of the intervention and control groups, 2.0% per year CVD event rate in the control group, 25% risk reduction associated with the intervention, average follow-up duration of three years, intra-cluster correlation coefficient (ICC) within villages of 0.025 for CVD events, two-sided significance level of 0.05, and statistical power of 90%. The overall minimum sample size is 32,000 subjects (16,000 in each comparison group and 100 participants in each cluster)

based on the Farrington & Manning Score test.[38] Loss to follow-up is not taking into consideration in sample size estimate due to availability of study villages and participants. However, we may elect to extend follow-up of participants for one more year if needed. For phase 1, we have >99.9% statistical power to detect a 10% difference in the primary outcome between the two comparison groups assuming 20% of participants in the control and 30% in the intervention group achieve BP <130/80 mmHg at 18 months. An ICC of 0.05 for hypertension control is assumed.[18] The statistical power is calculated using a Z test with a two-sided significance level of 0.05.[37]

We also calculated statistical power for phase 3 based on the following assumptions: 163 clusters in each group, 104 participants per cluster, a 5.0% proportion of dementia over 4 years, a 15% risk reduction, an average follow-up duration of 4 years, a lost-to-follow up rate of 1.6% per year, an intra-cluster correlation of 0.001, and a 2-sided significance level of 0.05. Dementia event is based on previous studies from Chinese population[39,40] and effect size is based on the experience from SPRINT-MIND and other previous trials.[41] The overall statistical power was determined to be 85.8%. The test statistic used is the two-sided Score test (Farrington & Manning) and implemented using PASS software.[38]

16. Data Management

BP readings are directly transferred from BP monitors to a central data system using a wireless network. All other data are double entered or transmitted directly from study sites to the central study database in the Department of Cardiology at the First Hospital of China Medical University, Liaoning, China. Study forms with missing values or errors are sent back to field study staff for correction. Two independent databases will be sent to the Department of Epidemiology at Tulane University for a final data check and quality control.

17. Data Analysis Plan

Intention-to-treat analyses will be conducted, in which study outcomes will be compared between participants according to their village randomization assignment, regardless of their actual adherence to the intervention. A 2-sided p-value <0.05 will be considered statistically significant.

In phase 1, we will test the difference in the proportions of patients with controlled BP between the two comparison groups using a generalized linear mixed-effects model:[42,43] $\log [P_{ij} /(1 - P_{ij})] = b0 + b1G_j + b2_j$, where P_{ij} denotes the probability of controlled BP for the ith participant, in the jth village at 18 months. The binary variable G_j indicates which randomization group is assigned to the jth village. The fixed effect b1 represents the intervention effect-the log-odds ratio of controlled BP associated with the intervention. $b2_j$ are the random effects at the village level with $b2_j \sim N(0, \upsilon^2)$, and the correlations of the outcome among the participants within each village are accounted for by assuming a compound symmetric correlation structure.

In addition, the difference in mean BP changes between the intervention and control groups will be tested using a linear mixed-effects model [42,43]. $Y_{ijl} = a0 + a1G_j + a2T_{ijl} + b1_j + b2_{ij} + e_{ijl}$, where Y_{ijl} is BP change from baseline for the ith participant nested in the jth village at the lth time. The fixed effect a1 represents the overall intervention effect across the study period, and a2 represents the time effect. We assume multilevel (nested) random effects at both village and patient levels, and they are represented by $b1_j$ with $b1_j \sim N(0, \upsilon^2)$ and $b2_{ij}$ with $b2_{ij} \sim N(0, \tau^2)$, respectively. The error, e_{ijl}, follows $N(0, \sigma^2)$. Rejection of the null hypothesis, a1=0, indicates a significant difference in BP change between the two comparison groups. Although an autoregressive correlation structure is the logical choice for these repeated measures, other correlation structures will be investigated as well.

In phase 2, the cumulative CVD event rates will be calculated using time-to-event methods according to randomization group and stratified by villages. The difference will be tested using a clustered log-rank test with the null hypothesis that cumulative incidences are the same between the two comparison groups.[44] Marginal structural Cox proportional hazards models will be used to assess the effectiveness of village doctor-led intervention vs. control on CVD event rates stratified by village.[45,46] The hazard ratios and 95% confidence intervals associated with intervention effect will be presented.

Co-variables will not be adjusted in the primary analysis. In a sensitivity analysis, important co-variables such as age, sex, education, duration of hypertension, and unbalanced CVD risk factors between the two comparison groups will be adjusted. Results from primary and sensitivity analyses will be compared. If they are consistent, the findings are less likely to be due to potential bias. In a sensitivity analysis, we will conduct multiple imputations for missing data, and the findings from these analyses will be compared to those from the primary analysis without imputation.[47]

In phase 3, the proportions of all-cause dementia and secondary outcomes will be calculated according to the randomization groups. In this study, it is not possible to estimate the exact time of dementia onset because cognitive function tests are only conducted at the 48-month follow-up visit. Log-binomial and robust (or modified) Poisson regression models are most frequently applied to estimate the risk ratio for binary outcomes. [48,49] Compared to the log-binomial model, robust Poisson regression provides unbiased estimates when the link function is mis-specified or when the probability distribution of the response variable is truncated at the right tail.[50] In this trial, the risk ratio of village doctor-led intervention versus control on dementia event rates will be estimated using robust Poisson regression with a robust error variance, stratified by village, town, county, and province.[49] SAS Proc GENMOD will be used for Poisson regression analysis.

Co-variables will not be adjusted in the primary analysis. In a sensitivity analysis, important co-variables such as age, sex, education, cigarette smoking, history of major cardiovascular disease, use of antihypertensive medication, systolic blood pressure, low-density lipoprotein cholesterol, and fasting

plasma glucose at baseline will be adjusted. In addition, predefined subgroup analyses by age, sex, education, antihypertensive medication, and ASCVD risk in phases 1 and 2; and by age, sex, education, cigarette smoking, body-mass index, systolic BP, fasting plasma glucose, and risk for 10-year ASCVD in phase 3 will be conducted. The Bonferroni correction method will be used to adjust the critical value for interaction tests in the subgroup analyses.[51]

18. Quality Control

Quality control will be conducted by a team of investigators, key research staff, and project inspectors. Strict quality control will be implemented at every step of the study including project preparation, training, screening, intervention, and data collection. A manual of procedures will be developed to detail the standardized approaches used in the study, such as participant recruitment, intervention, village doctor training (protocol-based treatment, health coaching, and follow-up), patient education (self-BP monitoring methods, lifestyle changes, and medication adherence), and other procedures of the study. All study personnel will be required to participate in a study training session prior to the initiation of any study procedures. The quality control team will review the study data regularly to ensure that all phases of the project are strictly implemented according to the study protocol and the authenticity, completeness, accuracy and reliability of the research data.

18.1. Preparation phase

A manual of procedures will include detailed descriptions of all trial procedures and will be used for training purposes and as a reference for all study investigators and staff. Standard forms, devices, and procedures in the field for BP measurement and other data collection procedures will be standardized. Furthermore, standard event definitions and event validation procedures will be used. The project will purchase the devices used in the study which pass the national quality inspection and provide these to local research teams. We will calibrate each device before they are distributed to study sites. The study protocol, manual of procedures, study forms, training materials and other written materials will be prepared centrally and sent to each study site.

18.2. Training phase

18.2.1. Research staff training

- We will hire research staff who can commit to the project for more than 3 years with a strong sense of responsibility and good communication skills.
- A standard training program will be developed, and all research staff will be required to attend centralized training.
- Regional retraining will be arranged in advance and all training materials will be prepared by the data coordinating center.

- All research staff will be certified prior to field work.

18.2.2. Village doctor training

- All village doctors in the intervention group will attend a one-day in-person session and on-site certification.
  - standard BP measurement methods
  - protocol-based antihypertensive management
  - health coaching on lifestyle modifications
  - medication side effects and adherence to the intervention
  - tracking and managing patients using the internet and mobile devices
- Retraining and quality improvement consultations will occur frequently in the first six months and once every six months afterwards.

18.3. Screening and recruitment phase

Screening and patient recruitment will be conducted in each village by the research staff and assisted by village doctors.

- Prepare all study forms and devices before the screening begins.
- Calibrate BP monitors and weigh scales regularly.
- During the screening, quality control staff in field will observe all procedures, including questionnaire interview and BP measurement. They will identify problems and correct them in a timely manner. They will double check a random sample of study forms to make sure these meet the study requirements.
- Quality control will also be conducted at the data coordinating center and data quality issues will be promptly feedbacked to field team.

18.4. Study conduct phase

18.4.1. Intervention

- All village doctors in the intervention group will be trained and certified.
- Village doctors will meet with primary care physicians from township hospital or CDC monthly to discuss and solve problems related to patient management.
- Quality control staff will randomly select study participants and assess the frequency and quality of village doctors' intervention performance. This information will be feedbacked to village

doctors for improvement.

18.4.2. Data collection

- All study data will be collected by trained and certified research staff from universities and major medical centers.
- During data collection, quality control staff in field will double check a random sample of study forms to make sure these meet the study requirements.
- Quality control will also be conducted at the data coordinating center and data quality issues will be promptly feedbacked to field team.
- Retraining will be conducted every six months. If research staff cannot meet criteria for data quality, they will be replaced.

19. Safety Monitoring

19.1. Adverse events and reporting

In this implementation trial, village doctors have the primary responsibility for the safety of the patients under their care. Village doctors will report all serious adverse events (SAEs) to the Coordinating Center. By definition, SAEs are adverse events that meet any of the following criteria:

- fatal or life-threatening,
- result in significant or persistent disability,
- require or prolong hospitalization,
- result in a congenital anomaly/birth defect, or
- are important medical events that investigators judge to represent significant hazards or harm to research participants and may require medical or surgical intervention to prevent one of the other outcomes listed in this definition (e.g. hospitalization, death, persistent disability).

At the follow-up visits, the research staff will specifically query participants for below adverse effects

- hypotension
- injurious falls
- syncope
- unexpected events for which the investigator believes that antihypertensive intervention caused the event or contributed to the immediate cause of the event

19.2. Data safety and monitoring board (DSMB)

An independent DSMB will be established to monitor data and oversee participant safety. Members will be appointed by the IRB of the First Hospital of China Medical University. The DSMB will include experts in hypertension, cardiology, neurology, clinical trials, and biostatistics. The DSMB normally meets once a year to monitor safety, to advise the investigator team about study progress and performance, and to make recommendations to China Medical University regarding study continuation and protocol changes. Before each meeting, the Coordinating Center will prepare and provide data on SAEs and selected AEs and any other safety information requested by the DSMB for discussion during open and closed sessions of DSMB meetings. The DSMB members will vote during the closed sessions on recommendations to continue or terminate the study based on safety data.

20. Study Organization

The CRHC project is led by investigators in the First Hospital of China Medical University and Tulane University in collaboration with investigators at the First Affiliated Hospital of Medical College, Xi'an Jiaotong University, Tongji Hospital, Tongji Medical College of Huazhong University of Science and Technology, and other Hospitals and CDCs in Liaoning, Hubei, and Shaanxi provinces, China.

20.1. Study Steering Committee

The study will be overseen by a Steering Committee, which will be chaired by the Principal Investigator (PI) and consist of investigators from the Study and Data Coordinating Center, and from each Field Coordinating Center. The Steering Committee will oversee all operations of the proposed study (**Figure 3**).

- Provide leadership and scientific oversight for the study
- Develop and approve the study protocol, intervention program, manual of operation, and study forms
- Oversee the selection of study sites and villages
- Monitor patient recruitment and retention, intervention delivery, and data collection
- Oversee study quality assurance and quality control
- Oversee data analyses, presentations, publications, and dissemination of study findings

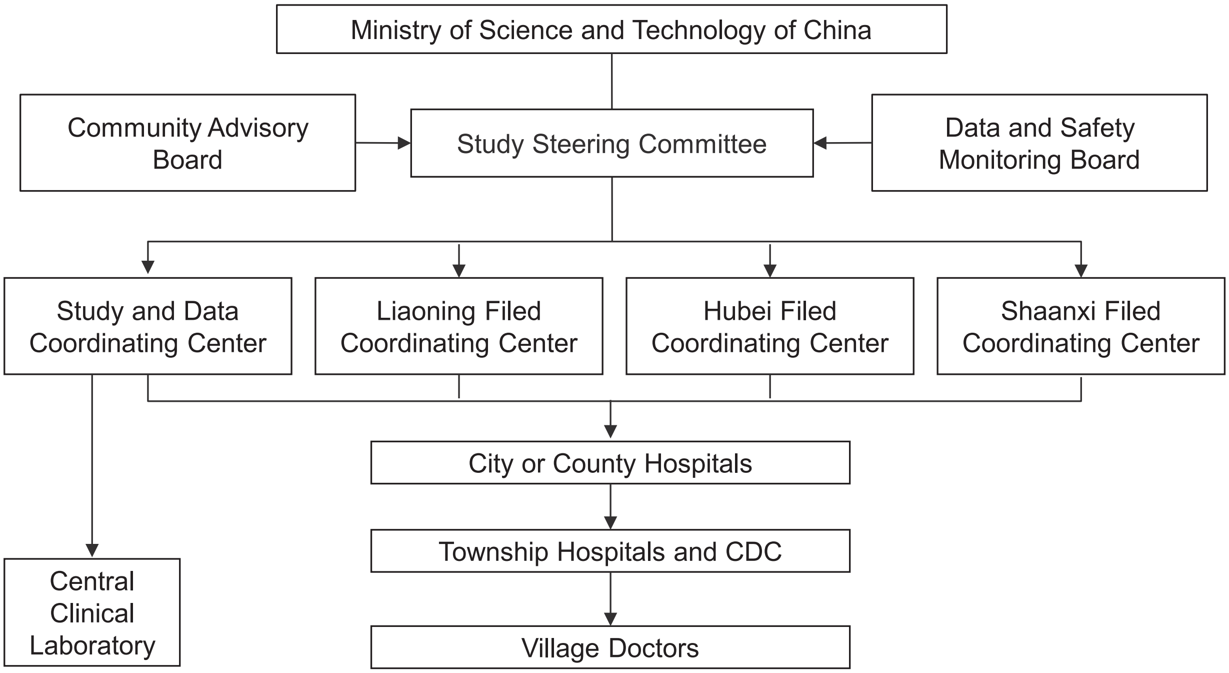

Figure 3. Study Organization Structure of China Rural Hypertension Control Project

The Steering Committee will meet annually in-person and monthly by conference call to discuss and make decisions on important study-related scientific and management issues. Important information, such as recruitment yields, data completion and quality, and adherence to the protocol, will be sent to committee members and core staff monthly. In addition, investigators will use e-mail communication frequently.

20.2. Study and Data Coordinating Center

The Study and Data Coordinating Center will be located at the First Hospital of China Medical University and Tulane University Translational Science Institute. The coordinating center will coordinate efforts on study protocol development, IRB approval, randomization, study conduct, data management, outcome adjudication, statistical analysis, and study finding dissemination.

- Develop and finalize the study protocol, study forms, and manual of procedures
- Prepare informed consents and the IRB application and obtain IRB approval for the study protocol and related materials
- Design and maintain a mobile BP management system
- Design and maintain an internet-based database management system
- Conduct randomization of study villages

- Arrange training and re-training for research staff on the data collection
- Arrange village doctor training on intervention
- Monitor patient recruitment, adherence, and retention and generate related data for the Study Steering Committee
- Organize the Study Steering Committee and DSMB meetings
- Coordinate study outcome adjudication activities
- Meet all regulatory requirements, such as tracking and reporting any adverse events
- Organize and conduct study site visits
- Data quality control, including data auditing
- Conduct data analysis and coordinate study presentations and publications

20.3. Clinical laboratory

The central clinical laboratory will be responsible for properly receiving, storing and analyzing blood specimens.

- Receive, process, and store the transferred blood samples
- Follow standard procedures for clinical biochemical index testing
- Transmit all laboratory data to the Study and Data Coordinating Center on a regular basis

20.4. Field Coordination Center

Each Field Coordination Center will oversee a network of research teams at city and county hospitals as well as primary care physicians at township hospitals/CDCs and village doctors within each province. Field coordinating centers will coordinate the day-to-day operations of the project in each province to ensure efficient and standardized trial performance. Its main responsibilities include:

- Work with the coordinating center to select study sites and villages
- Work with the coordinating center to conduct training and certification for research team and village doctors
- Coordinate and supervise each site during recruitment, intervention, data collection, and follow-up.
- Conduct regular site visits and identify and solve problems promptly
- Coordinate collection, temporary storage, and transportation of the blood samples to central laboratory

- Organize investigators and staff meetings regularly to facilitate communication within research team
- Responsible for the planning and management of project funds

21. Ethical Considerations

This study will be performed in accordance with the Declaration of Helsinki and evidence-based clinical practice guidelines. The trial has been approved by the Institutional Review Board of the First Hospital of China Medical University and all participating institutes. Informed consent is signed by all participants during screening visit. If a participant is unable to read or understand the informed consent, the research staff will read and explain it and give patient sufficient time to consider participation.

22. Patient Management during the COVID-19 Outbreak

In January 2020, coronavirus disease 2019 (COVID-19) outbreak occurred in Wuhan and rapidly spread to other regions in China. Many cities were locked down and transportation was restricted. One of our field coordinating centers is in Wuhan, Hubei Province. It is not possible to conduct in-person intervention and follow-up visits during the COVID-19 pandemic. Therefore, the Study Steering Committee and DSMB approves the following amendment to the study protocol.

- The study staff should actively and timely contact study participants in Hubei Province and directly mail antihypertensive medications to the patients to ensure their continuous access to antihypertensive medications
- Village doctors should conduct telephone visits with patients. Mean of self-measured BP from multiple days and information on medication compliance will be used to adjust antihypertensive medication
- Health coaching and follow-up visits will also be conducted by telephone

In addition, these COVID-19 measures could be applied to other field centers if COVID-19 lockdowns occur.

23. Dissemination and Scale-up

Barriers for implementing and maintaining village doctor-led projects for chronic disease management, including lack of official and professional support, insufficient training, and inadequate incentives, have been widely reported. The proposed village doctor-led multifaceted intervention is designed to overcome these barriers. Strong support from local health authorities, integration with the existing healthcare system, training and professional consultation, and financial support and incentives will empower village doctors for hypertension management. The CRHC project will fill the implementation knowledge gap for hypertension control in rural China. If proven effective, village doctor-led multifaceted intervention

programs could be scaled-up nationwide in China and other low- and middle-income countries to improve hypertension control and reduce the CVD and all-cause mortality burdens.

24. Timeline

The CRHC Project will last for five years. Timeline and tasks are presented in **Table 3**.

Table 3. Timeline and tasks

Tasks	2017		2018				2019				2020				2021				2022				2023			
	Q3	Q4	Q1	Q2	Q3	Q4	Q1	Q2	Q3	Q4	Q1	Q2	Q3	Q4	Q1	Q2	Q3	Q4	Q1	Q2	Q3	Q4	Q1	Q2	Q3	Q4
Study protocol, MOP, and study instruments	■	■	■																							
Communication with local authority		■	■																							
Needs assessment survey of village doctors			■																							
Study preparation and training			■																							
Pilot testing			■																							
Recruitment and baseline data collection				■	■	■																				
Intervention and data collection				■	■	■	■	■	■	■	■	■	■	■	■	■	■	■	■	■	■	■				
Data entry and quality control				■	■	■	■	■	■	■	■	■	■	■	■	■	■	■	■	■	■	■				
Analysis and manuscript preparation															■	■	■	■	■	■	■	■	■	■	■	■

25. References

1.He J, Gu D, Chen J, Wu X, Kelly TN, Huang JF, Chen JC, Chen CS, Bazzano LA, Reynolds K, Whelton PK and Klag MJ. Premature deaths attributable to blood pressure in China: a prospective cohort study. *Lancet*. 2009;374:1765-1772.

2. Li Y, Yang L, Wang L, Zhang M, Huang Z, Deng Q, Zhou M, Chen Z and Wang L. Burden of hypertension in China: A nationally representative survey of 174,621 adults. *Int J Cardiol*. 2017;227:516-523.

3. Wang Z, Chen Z, Zhang L, Wang X, Hao G, Zhang Z, Shao L, Tian Y, Dong Y, Zheng C, Wang J, Zhu M, Weintraub WS and Gao R. Status of Hypertension in China: Results From the China Hypertension Survey, 2012-2015. *Circulation*. 2018;137:2344-2356.

4. Wang J, Zhang L, Wang F, Liu L and Wang H. Prevalence, awareness, treatment, and control of hypertension in China: results from a national survey. *Am J Hypertens*. 2014;27:1355-1361.

5. Li W, Gu H, Teo KK, Bo J, Wang Y, Yang J, Wang X, Zhang H, Sun Y, Jia X, He X, Zhao X, Cheng X, Li J, Rangarajan S, Chen C, Yusuf S and Liu L. Hypertension prevalence, awareness, treatment, and control in 115 rural and urban communities involving 47 000 people from China. *J Hypertens*. 2016;34:39-46.

6. Lu J, Lu Y, Wang X, Li X, Linderman GC, Wu C, Cheng X, Mu L, Zhang H, Liu J, Su M, Zhao H, Spatz ES, Spertus JA, Masoudi FA, Krumholz HM and Jiang L. Prevalence, awareness, treatment, and control of hypertension in China: data from 1·7 million adults in a population-based screening study (China PEACE Million Persons Project). *Lancet*. 2017;390:2549-2558.

7. Chen WW, Gao RL, Liu LS, Zhu ML, Wang W, Wang YJ, Wu ZS, Li HJ, Gu DF, Yang YJ, Zheng Z, Jiang LX, Hu SS. China cardiovascular diseases report 2015: a summary. *J Geriatr Cardiol*. 2017;14:1-10.

8. Lewington S, Clarke R, Qizilbash N, Peto R and Collins R. Age-specific relevance of usual blood pressure to vascular mortality: a meta-analysis of individual data for one million adults in 61 prospective studies. *Lancet*. 2002;360:1903-13.

9. Ettehad D, Emdin CA, Kiran A, Anderson SG, Callender T, Emberson J, Chalmers J, Rodgers A and Rahimi K. Blood pressure lowering for prevention of cardiovascular disease and death: a systematic review and meta-analysis. *Lancet*. 2016;387:957-967.

10. Wright JT, Jr., Williamson JD, Whelton PK, Snyder JK, Sink KM, Rocco MV, Reboussin DM,

Rahman M, Oparil S, Lewis CE, Kimmel PL, Johnson KC, Goff DC, Jr., Fine LJ, Cutler JA, Cushman WC, Cheung AK and Ambrosius WT. A Randomized Trial of Intensive versus Standard Blood-Pressure Control. *N Engl J Med*. 2015;373:2103-16.

11. Bundy JD, Li C, Stuchlik P, Bu X, Kelly TN, Mills KT, He H, Chen J, Whelton PK and He J. Systolic Blood Pressure Reduction and Risk of Cardiovascular Disease and Mortality: A Systematic Review and Network Meta-analysis. *JAMA Cardiol*. 2017;2:775-781.

12. Whelton PK, Carey RM, Aronow WS, Casey DE, Jr., Collins KJ, Dennison Himmelfarb C, DePalma SM, Gidding S, Jamerson KA, Jones DW, MacLaughlin EJ, Muntner P, Ovbiagele B, Smith SC, Jr., Spencer CC, Stafford RS, Taler SJ, Thomas RJ, Williams KA, Sr., Williamson JD and Wright JT, Jr. 2017 ACC/AHA/AAPA/ABC/ACPM/AGS/APhA/ASH/ASPC/NMA/PCNA Guideline for the Prevention, Detection, Evaluation, and Management of High Blood Pressure in Adults: Executive Summary: A Report of the American College of Cardiology/American Heart Association Task Force on Clinical Practice Guidelines. *J Am Coll Cardiol*. 2018;71:2199-2269.

13. Writing Group of the 2010 Chinese Guidelines for the Management of Hypertension. 2010 Chinese guidelines for the management of hypertension. Chin J Cardiol. 2011;39:579-615 (Chinese).

14. Writing Group of the 2018 Chinese Guidelines for the Management of Hypertension. 2018 Chinese guidelines for the management of hypertension. Chin J Cardiovasc Med. 2019;24:24-56 (Chinese).

15. Huang W, Long H, Li J, Tao S, Zheng P, Tang S, Abdullah AS. Delivery of public health services by community health workers (CHWs) in primary health care settings in China: a systematic review (1996-2016). *Glob Health Res Policy*. 2018;3:18.

16. Long H, Huang W, Zheng P, Li J, Tao S, Tang S, Abdullah AS. Barriers and Facilitators of Engaging Community Health Workers in Non-Communicable Disease (NCD) Prevention and Control in China: A Systematic Review (2006‒2016). *Int J Environ Res Public Health*. 2018;15:2378.

17. Mills KT, Obst KM, Shen W, Molina S, Zhang HJ, He H, Cooper LA, He J. Comparative Effectiveness of Implementation Strategies for Blood Pressure Control in Hypertensive Patients: A Systematic Review and Meta-analysis. *Ann Intern Med*. 2018;168:110-120.

18. He J, Irazola V, Mills KT, Poggio R, Beratarrechea A, Dolan J, Chen CS, Gibbons L, Krousel-Wood M, Bazzano LA, Nejamis A, Gulayin P, Santero M, Augustovski F, Chen J, Rubinstein A; HCPIA Investigators. Effect of a Community Health Worker-Led Multicomponent Intervention on Blood Pressure Control in Low-Income Patients in Argentina: A Randomized Clinical Trial. *JAMA*. 2017;318:1016-1025.

19. Khatib R, Schwalm JD, Yusuf S, Haynes RB, McKee M, Khan M, Nieuwlaat R. Patient and healthcare provider barriers to hypertension awareness, treatment and follow up: a systematic review and meta-analysis of qualitative and quantitative studies. PLoS One. 2014;9(1):e84238.

20. Pickering TG, Hall JE, Appel LJ, Falkner BE, Graves JW, Hill MN, Jones DH, Kurtz T, Sheps SG and Roccella EJ. Recommendations for blood pressure measurement in humans: an AHA scientific statement from the Council on High Blood Pressure Research Professional and Public Education Subcommittee. *J Clin Hypertens (Greenwich)*. 2005;7:102-109.

21. Thygesen K, Alpert JS, Jaffe AS, Simoons ML, Chaitman BR, White HD; Joint ESC/ACCF/AHA/WHF Task Force for Universal Definition of Myocardial Infarction; Authors/Task Force Members Chairpersons, Thygesen K, Alpert JS, White HD; Biomarker Subcommittee, Jaffe AS, Katus HA, Apple FS, Lindahl B, Morrow DA; ECG Subcommittee, Chaitman BR, Clemmensen PM, Johanson P, Hod H; Imaging Subcommittee, Underwood R, Bax JJ, Bonow JJ, Pinto F, Gibbons RJ; Classification Subcommittee, Fox KA, Atar D, Newby LK, Galvani M, Hamm CW; Intervention Subcommittee, Uretsky BF, Steg PG, Wijns W, Bassand JP, Menasche P, Ravkilde J; Trials & Registries Subcommittee, Ohman EM, Antman EM, Wallentin LC, Armstrong PW, Simoons ML; Trials & Registries Subcommittee, Januzzi JL, Nieminen MS, Gheorghiade M, Filippatos G; Trials & Registries Subcommittee, Luepker RV, Fortmann SP, Rosamond WD, Levy D, Wood D; Trials & Registries Subcommittee, Smith SC, Hu D, Lopez-Sendon JL, Robertson RM, Weaver D, Tendera M, Bove AA, Parkhomenko AN, Vasilieva EJ, Mendis S; ESC Committee for Practice Guidelines (CPG), Bax JJ, Baumgartner H, Ceconi C, Dean V, Deaton C, Fagard R, Funck-Brentano C, Hasdai D, Hoes A, Kirchhof P, Knuuti J, Kolh P, McDonagh T, Moulin C, Popescu BA, Reiner Z, Sechtem U, Sirnes PA, Tendera M, Torbicki A, Vahanian A, Windecker S; Document Reviewers, Morais J, Aguiar C, Almahmeed W, Arnar DO, Barili F, Bloch KD, Bolger AF, Botker HE, Bozkurt B, Bugiardini R, Cannon C, de Lemos J, Eberli FR, Escobar E, Hlatky M, James S, Kern KB, Moliterno DJ, Mueller C, Neskovic AN, Pieske BM, Schulman SP, Storey RF, Taubert KA, Vranckx P, Wagner DR. Third universal definition of myocardial infarction. J Am Coll Cardiol. 2012;60(16):1581-98.

22. World Health Organization. International classification of diseases. 10th revision. Geneva: World Health Organization, 1993.

23. Mohr JP, Thompson JL, Lazar RM, Levin B, Sacco RL, Furie KL, Kistler JP, Albers GW, Pettigrew LC, Adams HP Jr, Jackson CM, Pullicino P; Warfarin-Aspirin Recurrent Stroke Study Group. A comparison of warfarin and aspirin for the prevention of recurrent ischemic stroke. N Engl J Med. 2001; 345(20):1444-51.

24. Ay H, Benner T, Arsava EM, Furie KL, Singhal AB, Jensen MB, Ayata C, Towfighi A, Smith EE, Chong JY, Koroshetz WJ, Sorensen AG. A computerized algorithm for etiologic classification of ischemic stroke - The causative classification of stroke system: Stroke. 2007; 38:2979-2984.

25. Rosamond WD, Chang PP, Baggett C, Johnson A, Bertoni AG, Shahar E, Deswal A, Heiss G, Chambless LE. Classification of heart failure in the atherosclerosis risk in communities (ARIC) study: a comparison of diagnostic criteria. *Circ Heart Fail*. 2012;5(2):152-159.

26. Rosamond WD, Chang P, Baggett C, Bertoni A, Shahar E, Deswal A, Heiss G, Chambless L. Classification of Heart Failure in the Atherosclerosis Risk in Communities (ARIC) Study: A Comparison With Other Diagnostic Criteria: Circulation. 2009; 120:S506.

27. Hiratzka LF, Bakris GL, Beckman JA, Bersin RM, Carr VF, Casey DE Jr, Eagle KA, Hermann LK, Isselbacher EM, Kazerooni EA, Kouchoukos NT, Lytle BW, Milewicz DM, Reich DL, Sen S, Shinn JA, Svensson LG, Williams DM; American College of Cardiology Foundation/American Heart Association Task Force on Practice Guidelines; American Association for Thoracic Surgery; American College of Radiology; American Stroke Association; Society of Cardiovascular Anesthesiologists; Society for Cardiovascular Angiography and Interventions; Society of Interventional Radiology; Society of Thoracic Surgeons; Society for Vascular Medicine. 2010 ACCF/AHA/AATS/ACR/ASA/SCA/SCAI/SIR/STS/SVM Guidelines for the diagnosis and management of patients with thoracic aortic disease. A Report of the American College of Cardiology Foundation/American Heart Association Task Force on Practice Guidelines, American Association for Thoracic Surgery, American College of Radiology,American Stroke Association, Society of Cardiovascular Anesthesiologists, Society for Cardiovascular Angiography and Interventions, Society of Interventional Radiology, Society of Thoracic Surgeons,and Society for Vascular Medicine. *J Am Coll Cardiol*. 2010;55(14):e27-e129.

28. Folstein MF, Folstein SE, McHugh PR. Mini-mental state: A practical method for grading the cognitive state of patients for the clinician. J Psychiatr Res. 1975 Nov;12(3):189-98.

29. Katzman R, Zhang MY, Ouang-Ya-Qu, Wang ZY, Liu WT, Yu E, Wong SC, Salmon DP, Grant I. A Chinese version of the Mini-Mental State Examination; impact of illiteracy in a Shanghai dementia survey. J Clin Epidemiol. 1988;41(10):971-8. doi: 10.1016/0895-4356(88)90034-0.

30. Li H, Jia J, Yang Z. Mini-Mental State Examination in Elderly Chinese: A Population-Based Normative Study. J Alzheimers Dis. 2016;53(2):487-96. doi: 10.3233/JAD-160119.

31. Pfeffer RI, Kurosaki TT, Harrah CH, Chance JH, Filos S. Measurement of functional activities in older adults in the community. J Gerontol. 1982;37(3):323-9.

32. González DA, Gonzales MM, Resch ZJ, Sullivan AC, Soble JR. Comprehensive Evaluation of the Functional Activities Questionnaire (FAQ) and Its Reliability and Validity. Assessment. 2022;29(4):748-763.

33. Yin L, Ren Y, Wang X, Li Y, Hou T, Liu K, Cong L, Zhang Q, Wang Y, Jiang Z, Du Y. The power of the Functional Activities Questionnaire for screening dementia in rural-dwelling older adults at high-risk of cognitive impairment. Psychogeriatrics. 2020;20(4):427-436.

34. Galvin JE. The Quick Dementia Rating System (QDRS): A Rapid Dementia Staging Tool. Alzheimers Dement (Amst). 2015 Jun 1;1(2):249-259.

35. Pang T, Chong EJY, Wong ZX, Chew KA, Venketasubramanian N, Chen C, Xu X. Validation of the Informant Quick Dementia Rating System (QDRS) among Older Adults in Singapore. J Alzheimers Dis. 2022;89(4):1323-1330.

36. McKhann GM, Knopman DS, Chertkow H, Hyman BT, Jack CR Jr, Kawas CH, Klunk WE, Koroshetz WJ, Manly JJ, Mayeux R, Mohs RC, Morris JC, Rossor MN, Scheltens P, Carrillo MC, Thies B, Weintraub S, Phelps CH. The diagnosis of dementia due to Alzheimer's disease: recommendations from the National Institute on Aging-Alzheimer's Association workgroups on diagnostic guidelines for Alzheimer's disease. Alzheimers Dement. 2011;7(3):263-9.

37. Albert MS, DeKosky ST, Dickson D, Dubois B, Feldman HH, Fox NC, Gamst A, Holtzman DM, Jagust WJ, Petersen RC, Snyder PJ, Carrillo MC, Thies B, Phelps CH. The diagnosis of mild cognitive impairment due to Alzheimer's disease: recommendations from the National Institute on Aging-Alzheimer's Association workgroups on diagnostic guidelines for Alzheimer's disease. Alzheimers Dement 2011;7(3): 270-9.

38. Donner A, Klar N. Design and Analysis of Cluster Randomization Trials in Health Research. London: Arnold, 2000.

39. Yuan J, Zhang Z, Wen H, Hong X, Hong Z, Qu Q, Tang M, Wu J, Xu Q, Li H, Cummings JL. Incidence of dementia and subtypes: A cohort study in four regions in China. Alzheimers Dement. 2016 Mar;12(3):262-71.

40. Jia L, Quan M, Fu Y, Zhao T, Li Y, Wei C, Tang Y, Qin Q, Wang F, Qiao Y, Shi S, Wang YJ, Du Y, Zhang J, Zhang J, Luo B, Qu Q, Zhou C, Gauthier S, Jia J; Group for the Project of Dementia Situation in

China. Dementia in China: epidemiology, clinical management, and research advances. Lancet Neurol. 2020 Jan;19(1):81-92.

41. SPRINT MIND Investigators for the SPRINT Research Group; Williamson JD, Pajewski NM, Auchus AP, Bryan RN, Chelune G, Cheung AK, Cleveland ML, Coker LH, Crowe MG, Cushman WC, Cutler JA, Davatzikos C, Desiderio L, Erus G, Fine LJ, Gaussoin SA, Harris D, Hsieh MK, Johnson KC, Kimmel PL, Tamura MK, Launer LJ, Lerner AJ, Lewis CE, Martindale-Adams J, Moy CS, Nasrallah IM, Nichols LO, Oparil S, Ogrocki PK, Rahman M, Rapp SR, Reboussin DM, Rocco MV, Sachs BC, Sink KM, Still CH, Supiano MA, Snyder JK, Wadley VG, Walker J, Weiner DE, Whelton PK, Wilson VM, Woolard N, Wright JT Jr, Wright CB. Effect of Intensive vs Standard Blood Pressure Control on Probable Dementia: A Randomized Clinical Trial.. JAMA. 2019;321(6):553-561.

42. Brown H, Prescott R. Applied Mixed Models in Medicine, 3rd Edition. New York: John Wiley & Sons. 2015.

43. Diggle PJ, Liang KY, Zeger SL. Analysis of Longitudinal Data, 2nd Edition. Oxford: Oxford University Press, 2002.

44. Jung SH, Jeong JH. Rank tests for clustered survival data. *Lifetime Data Anal*. 2003;9:21-33.

45. Cox DR. Regression models and life tables (with discussion). Journal of the Royal Statistical Society. 1972; Series B 34, 187-220.

46. Lu SE and Wang MC. Marginal analysis for clustered failure time data. *Lifetime Data Anal*. 2005;11:61-79.

47. Bondarenko I and Raghunathan T. Graphical and numerical diagnostic tools to assess suitability of multiple imputations and imputation models. *Stat Med*. 2016;35:3007-3020.

48. Fitzmaurice GM, Lipsitz SR, Arriaga A, Sinha D, Greenberg C, Gawande AA. Almost efficient estimation of relative risk regression. Biostatistics. 2014;15(4):745-56.

49. Zou G. A modified Poisson regression approach to prospective studies with binary data. Am J Epidemiol. 2004;159(7):702-6.

50. Chen W, Qian L, Shi J, Franklin M. Comparing performance between log-binomial and robust Poisson regression models for estimating risk ratios under model misspecification. BMC Med Res Methodol. 2018;18, 63. https://doi.org/10.1186/s12874-018-0519-5.

51. Dunn OJ. Multiple Comparisons Among Means. J Am Stat Assoc. 1961;56(293):52–64.

China Rural Hypertension Control (CRHC) Project

Statistical Analysis Plan

November 2018
October 2020
October 2021

CRHC Project Study and Data Coordinating Center
The First Hospital of China Medical University, Shenyang, China
Tulane University Translational Science Institute, New Orleans, US

Section 1. Introduction

1. Background

Hypertension is the leading preventable risk factor for cardiovascular disease (CVD) and premature death in China.(1) The prevalence of hypertension is high and increasing while the control rate is low, especially in rural areas.(2-4) Uncontrolled hypertension plays an important role in the increased burden of CVD and stroke mortality in rural residents in China.(1) Traditionally, village doctors have played an important role in infectious disease control and delivering essential health services to rural residents in China.(5) With appropriate training and supervision, they could play an important role in hypertension control in rural China.(6)

2. Study Objectives

The overall objective of the CRHC Project is to develop an effective, feasible, and sustainable implementation strategy to achieve more intensive blood pressure (BP) control in rural residents in China. Moreover, this implementation trial will test the effectiveness of a lower BP target (<130/80 mmHg) on CVD outcomes. The specific aims for phases 1 and 2 of this project are:

Phase 1: to test whether a village doctor-led multifaceted intervention program will improve BP control (<130/80 mmHg) and reduce BP levels among hypertensive patients over an 18-month period compared to control.

Phase 2: to test whether a village doctor-led multifaceted intervention program will reduce CVD events, as well as mortality from CVD and all causes, among hypertensive patients over a 36-month period compared to control.

Phase 3: to test the effectiveness of a village doctor-led blood pressure intervention program compared to usual care on all-cause dementia and other cognitive impairment outcomes among patients with hypertension over a 48-month period.

Section 2. Study Methods

3. Study Design

The CRHC Project is a cluster randomized trial that will be conducted in 320 villages from three provinces in mainland China. One hundred and sixty villages will be randomly assigned to a village doctor-led multifaceted intervention and 160 villages to control, stratified by provinces, counties, and townships. A total of 32,000 individuals aged ≥40 years with uncontrolled hypertension will be recruited into the study. The village doctor-led multifaceted intervention is designed to overcome barriers at the healthcare system, provider, patient, and community levels.(7) Study participants are followed every 6 months for BP, CVD, and other study outcomes. The primary outcome is BP control (<130/80 mm Hg) at 18 months in phase 1, CVD events over 36 months in phase 2, and all-cause dementia over 48 months in phase 3.

4. Study Outcomes

Phase 1 will last for 18 months, phase 2 will last for 36 months, and phasse 3 will last for 48 minths. The primary and secondary study outcomes are as follows:

Phase 1 Study Outcomes

<u>Primary outcome:</u> Proportion of hypertension control (BP <130/80 mm Hg) at 18 months

<u>Secondary outcomes:</u>

- Changes in mean systolic and diastolic BP from baseline to 18 months
- Proportion of hypertension control (BP <140/90 mmHg)
- Proportion of patients adherent to antihypertensive medications at 18 months

Phase 2 Study Outcomes

Primary outcome: Composite cardiovascular disease (myocardial infarction, stroke, heart failure requiring hospitalization or treatment, and cardiovascular death)

Secondary outcome:

- Individual cardiovascular disease
  - Myocardial infarction
  - Stroke
  - Heart failure requiring hospitalization or treatment
  - Cardiovascular death
- All-cause mortality
- Changes in mean systolic and diastolic BP from baseline to 36 months
- Aortic dissection
- Incident malignant tumors

Phase 3 Study Outcomes

Primary outcome: All-cause dementia

Secondary outcome:

- Cognitive impairment no dementia
- Composite outcome of dementia or cognitive impairment no dementia
- Death from all causes
- Composite outcome of dementia or deaths
- Composite and individual cardiovascular disease (myocardial infarction, stroke, heart failure requiring hospitalization or treatment, and cardiovascular death)
- Changes in mean systolic and diastolic BP from baseline to 48 months
- Proportion of hypertension control (BP <130/80 mm Hg or <140/90 mmHg) at 48 months

5. Eligibility Criteria

Inclusion criteria for study villages:

- The village has a regular village doctor who is willing to participate in the hypertension control project
- The village does not plan to merge with other villages within 3 years
- The village is at least 2 kilometers away from other participating villages

- The village participates in the China New Rural Cooperative Medical Scheme

Inclusion criteria of study participants:

- Men or women aged ≥40 years
- Mean untreated systolic BP ≥140 mm Hg and/or diastolic BP ≥90 mm Hg or mean treated systolic BP ≥130 mm Hg and/or diastolic BP ≥80 mm Hg for individuals without a history of clinical CVD; or mean treated/untreated systolic BP ≥130 mm Hg and/or diastolic BP ≥80 mm Hg for individuals with a history of clinical coronary heart disease, heart failure, stroke, diabetes, or chronic kidney disease
- Have lived in a participating village for at least 6 months
- No intention to migrate within next 3 years
- Taking part in the New Rural Cooperative Medical Scheme
- Not pregnant or planning to become pregnant
- No malignant tumors and life expectancy ≥3 years
- Willing to participate and able to sign informed consent

6. Statistical Power and Sample Size

The sample size of the CRHC Project is calculated for the primary outcome of phase 2 due to the minimum required sample size being much larger in phase 2 than phase 1.

Phase 2 sample size:

The phase 2 sample size calculation is based on the following assumptions:

- 160 clusters in each of the intervention and control groups
- 2.0% per year CVD event rate in the control group
- 25% risk reduction associated with the intervention
- average follow-up duration of three years
- intra-cluster correlation coefficient (ICC) within villages of 0.025 for CVD events
- two-sided significance level of 0.05
- statistical power of 90%

The overall minimum sample size is 32,000 subjects (16,000 in each comparison group and 100 participants in each cluster) based on the Farrington & Manning Score test.(8) Loss to follow-up is not taking into consideration in sample size estimate due to availability of study villages and participants. However, we may elect to extend follow-up of participants for one more year if needed.

Phase 1 statistical power

Based on the sample size from phase 2, we have >99.9% statistical power to detect a 10% difference in the primary outcome between the two comparison groups based on the following assumptions:

- 20% of participants in the control group achieve BP <130/80 mmHg at 18 months
- 30% of participants in the intervention group achieve BP <130/80 mmHg at 18 months
- ICC of 0.05 for hypertension control.(9)

The statistical power is calculated using a Z test with a two-sided significance level of 0.05.(8)

Phase 3 statistical power

We calculated statistical power for phase 3 based on the following assumptions:

- 163 clusters in each group
- 104 participants per cluster
- 5.0% dementia event over 4 years
- 15% risk reduction
- average follow-up duration of 4 years
- lost-to-follow up rate of 1.6% per year
- intra-cluster correlation of 0.001, and
- 2-sided significance level of 0.05

Dementia event is based on previous studies from Chinese population (10,11) and effect size is based on a meta-analysis of results from the Syst-Eur (Systolic Hypertension in Europe) trial (12), the PROGRESS (Perindopril Protection Against Recurrent Stroke Study) trial (13), the HYVET (Hypertension in the Very Elderly) trial (14), and the SPRINT MIND (Systolic Blood Pressure Intervention Trial - Memory and Cognition in Decreased Hypertension) trial (15). The overall statistical power was determined to be 85.8%. The test statistic used is the two-sided Score test (Farrington & Manning) and implemented using PASS software (8).

Section 3. General Analysis Considerations

7. Timing of Analyses

The final analysis for phase 1 will be performed after all study participants have completed the 18-month visit 2; all study data have been transferred to the Study and Data Coordinating Center and passed quality control tests at the Tulane University Translational Science Institute; and this SAP document has been finalized and approved.

The final analysis for phase 2 will be performed after all study participants have completed the 36-month visit; all study data have been transferred to the Tulane University Translational Science Institute and documented as meeting the cleaning and quality requirements; and this SAP document has been finalized and approved.

The final analysis for phase 3 will be performed after all study participants have completed the 48-month visit; all study data have been transferred to the Tulane University Translational Science Institute and documented as meeting the cleaning and quality requirements; and this SAP document has been finalized and approved.

8. Analysis Populations

Intention-to-treat analyses will be conducted, in which study outcomes will be compared between participants according to their village randomization assignment, regardless of their actual adherence to the intervention.

9. Covariates and Subgroups

Co-variables will not be adjusted in the primary analysis. In a sensitivity analysis, important co-variables such as age, sex, education, duration of hypertension, and unbalanced CVD risk factors between the two comparison groups will be adjusted. Results from primary and sensitivity analyses will be compared. If they are consistent, the findings are less likely due to potential bias.

The pre-defined subgroup analyses will be conducted by the following covariables for Phase-1 and 2:

- age: <60 vs. ≥60 years
- sex: men vs. women
- education: <high school vs. ≥high school
- antihypertensive medication at baseline: use vs. no use
- cardiovascular risk: high risk (history of clinical myocardial infarction, stroke, or heart failure or atherosclerotic cardiovascular disease risk ≥20% based on ACC/AHA Pooled Population Equation) vs. low risk (<20%).(13)

The pre-defined subgroup analyses will be conducted by the following covariables for phase-3:

- age: median age of dementia participants
- sex: men vs. women
- education: median education level of dementia participants
- cigarette smoking: current smoking vs. not current smoking (never or former)
- body weight: median body mass index of dementia participants
- systolic blood pressure: median systolic blood pressure of dementia participants
- fasting plasma glucose: median fasting plasma glucose of dementia participants
- cardiovascular risk: median 10-year risk of atherosclerotic cardiovascular disease in dementia participants based on ACC/AHA Pooled Population Equation (16). Individuals with a history of clinical myocardial infarction, stroke, or heart failure will be categorized into the highest risk group.

10. Missing Data

In the primary analysis, no imputation will be used for missing data. In a secondary analysis, we will use multiple imputation for missing data in multivariable sensitivity analyses. The MCMC statement uses a Markov Chain Monte Carlo method to impute values for a data set with an arbitrary missing pattern, assuming a multivariate normal distribution for the data.(17,18) Point

estimates and confidence intervals will be compared from the complete case analysis and multiple imputation analysis.

11. Multiple Testing

The critical values will not be adjusted for in the primary and secondary study outcomes. There will be one primary study outcome in phase 1. Likewise, there will be one primary study outcome in phase 2 and phase 3.

The Bonferroni correction method will be used to adjust the critical value for interaction tests in the subgroup analyses. For example, a 2-sided p-value <0.003 (0.05/16) will be considered statistically significant for the ten tests in the subgroup analyses (19).

Section 4. Effectiveness and Other Analyses

12. Primary and Secondary Outcomes in Phase 1

In phase 1, we will test the difference in the proportions of patients with controlled BP between the two comparison groups using a generalized linear mixed-effects model:(20,21) $\log [P_{ij} /(1 - P_{ij})] = b0 + b1G_j + b2_j$, where P_{ij} denotes the probability of controlled BP for the ith participant, in the jth village at 18 months. The binary variable G_j indicates which randomization group is assigned to the jth village. The fixed effect b1 represents the intervention effect: the log-odds ratio of controlled BP associated with the intervention. $b2_j$ are the random effects at the village level with $b2_j \sim N(0, \upsilon^2)$, and the correlations of the outcome among the participants within each village are accounted for by assuming a compound symmetric correlation structure.

In addition, the difference in mean BP changes between the intervention and control groups will be tested using a linear mixed-effects model:(13,14) $Y_{ijl} = a0 + a1G_j + a2T_{ijl} + b1_j + b2_{ij} + e_{ijl}$, where Y_{ijl} is BP change from baseline for the ith participant nested in the jth village at the lth time. The fixed effect a1 represents the overall intervention effect across the study period, and a2 represents the time effect. We assume multilevel (nested) random effects at both village and patient levels, and they are represented by $b1_j$ with $b1_j \sim N(0, \upsilon^2)$ and $b2_{ij}$ with $b2_{ij} \sim N(0, \tau^2)$, respectively. The error, e_{ijl}, follows $N(0, \sigma^2)$. Rejection of the null hypothesis, $a1=0$, indicates a significant difference in BP change between the two comparison groups. Although an autoregressive correlation structure is the logical choice for these repeated measures, other correlation structures will be investigated as well.

13. Primary and Secondary Outcomes in Phase 2

In phase 2, the cumulative CVD event rates will be calculated using time-to-event methods according to randomization group and stratified by villages. The difference will be tested using a clustered log-rank test with the null hypothesis that cumulative incidences are the same between the two comparison groups.(22) Marginal structural Cox proportional hazards models will be used to assess the effectiveness of the village doctor-led intervention vs. control on CVD event rates stratified by village.(23,24) The hazard ratios and 95% confidence intervals associated with intervention effect will be presented.

Co-variables will not be adjusted in the primary analysis. In a sensitivity analysis, important co-variables such as age, sex, education, duration of hypertension, and unbalanced CVD risk factors between the two comparison groups will be adjusted. Results from the primary and sensitivity analyses will be compared. If they are consistent, the findings are less likely due to potential bias. In a sensitivity analysis, we will conduct multiple imputation for missing data, and

the findings from these analyses will be compared to those from the primary analysis without imputation.(25)

14. Primary and Secondary Outcomes in Phase 3

In phase 3, the proportions of all-cause dementia and secondary outcomes will be calculated according to the randomization group and stratified by township, county, and province. In this study, it is not possible to estimate the exact time of dementia onset because cognitive function tests are only conducted at the 48-month follow-up visit. Log-binomial and robust (or modified) Poisson regression models are most frequently applied to estimate the risk ratio for binary outcomes (26,27). Compared to the log-binomial model, robust Poisson regression provides unbiased estimates when the link function is mis-specified or when the probability distribution of the response variable is truncated at the right tail (28). In this trial, the risk ratio of village doctor-led intervention versus control on dementia event rates will be estimated using robust Poisson regression with a robust error variance, stratified by town, county, and province (28). Due to the clustered design, a compound symmetric working correlation structure will be utilized to account for the clustering effect within villages. SAS Proc GENMOD will be used for Poisson regression analysis.

Co-variables will not be adjusted in the primary analysis. In a sensitivity analysis, important co-variables such as age, sex, cigarette smoking, history of major cardiovascular disease, use of antihypertensive medication, systolic blood pressure, low-density lipoprotein cholesterol, and fasting plasma glucose at baseline will be adjusted.

15. Safety Analyses

Adverse events (AE) and serious adverse events (SAE) will be reported by comparison groups. Events will be reported as the number of and percentage of individuals experiencing an adverse event. Rates of adverse events will be compared using a χ^2 test.

16. Reference

1. He J, Gu D, Chen J, Wu X, Kelly TN, Huang JF, Chen JC, Chen CS, Bazzano LA, Reynolds K, Whelton PK, Klag MJ. Premature deaths attributable to blood pressure in China: a prospective cohort study. Lancet. 2009;374(9703):1765-72. Epub 2009/10/09. doi: 10.1016/s0140-6736(09)61199-5. PubMed PMID: 19811816.
2. Li Y, Yang L, Wang L, Zhang M, Huang Z, Deng Q, Zhou M, Chen Z, Wang L. Burden of hypertension in China: A nationally representative survey of 174,621 adults. Int J Cardiol. 2017;227:516-23. Epub 2016/11/20. doi: 10.1016/j.ijcard.2016.10.110. PubMed PMID: 27856040.
3. Lu J, Lu Y, Wang X, Li X, Linderman GC, Wu C, Cheng X, Mu L, Zhang H, Liu J, Su M, Zhao H, Spatz ES, Spertus JA, Masoudi FA, Krumholz HM, Jiang L. Prevalence, awareness, treatment, and control of hypertension in China: data from 1·7 million adults in a population-based screening study (China PEACE Million Persons Project). Lancet. 2017;390(10112):2549-58. Epub 2017/11/06. doi: 10.1016/s0140-6736(17)32478-9. PubMed PMID: 29102084.
4. Wang Z, Chen Z, Zhang L, Wang X, Hao G, Zhang Z, Shao L, Tian Y, Dong Y, Zheng C, Wang J, Zhu M, Weintraub WS, Gao R. Status of Hypertension in China: Results From the China Hypertension Survey, 2012-2015. Circulation. 2018;137(22):2344-56. Epub

2018/02/17. doi: 10.1161/circulationaha.117.032380. PubMed PMID: 29449338.

5. Li X, Lu J, Hu S, Cheng KK, De Maeseneer J, Meng Q, Mossialos E, Xu DR, Yip W, Zhang H, Krumholz HM, Jiang L, Hu S. The primary health-care system in China. Lancet. 2017;390(10112):2584-94. Epub 2017/12/13. doi: 10.1016/s0140-6736(17)33109-4. PubMed PMID: 29231837.

6. Long H, Huang W, Zheng P, Li J, Tao S, Tang S, Abdullah AS. Barriers and Facilitators of Engaging Community Health Workers in Non-Communicable Disease (NCD) Prevention and Control in China: A Systematic Review (2006⁻2016). Int J Environ Res Public Health. 2018;15(11). Epub 2018/10/31. doi: 10.3390/ijerph15112378. PubMed PMID: 30373205; PMCID: PMC6266440.

7. Sun Y, Li Z, Guo X, Zhou Y, Ouyang N, Xing L, Sun G, Mu J, Wang D, Zhao C, Wang J, Ye N, Zheng L, Chen S, Chang Y, Yang R, He J. Rationale and Design of a Cluster Randomized Trial of a Village Doctor-Led Intervention on Hypertension Control in China. Am J Hypertens. 2021;34(8):831-9. Epub 2021/02/20. doi: 10.1093/ajh/hpab038. PubMed PMID: 33605981.

8. Donner A, Klar N. Design and Analysis of Cluster Randomization Trials in Health Research. London: Arnold, 2000.

9. He J, Irazola V, Mills KT, Poggio R, Beratarrechea A, Dolan J, Chen CS, Gibbons L, Krousel-Wood M, Bazzano LA, Nejamis A, Gulayin P, Santero M, Augustovski F, Chen J, Rubinstein A. Effect of a Community Health Worker-Led Multicomponent Intervention on Blood Pressure Control in Low-Income Patients in Argentina: A Randomized Clinical Trial. Jama. 2017;318(11):1016-25. Epub 2017/10/05. doi: 10.1001/jama.2017.11358. PubMed PMID: 28975305; PMCID: PMC5761321.

10. Yuan J, Zhang Z, Wen H, Hong X, Hong Z, Qu Q, Tang M, Wu J, Xu Q, Li H, Cummings JL. Incidence of dementia and subtypes: A cohort study in four regions in China. Alzheimers Dement. 2016 Mar;12(3):262-71.

11. Jia L, Quan M, Fu Y, Zhao T, Li Y, Wei C, Tang Y, Qin Q, Wang F, Qiao Y, Shi S, Wang YJ, Du Y, Zhang J, Zhang J, Luo B, Qu Q, Zhou C, Gauthier S, Jia J; Group for the Project of Dementia Situation in China. Dementia in China: epidemiology, clinical management, and research advances. Lancet Neurol. 2020 Jan;19(1):81-92.

12. Forette F, Seux ML, Staessen JA, Thijs L, Birkenhäger WH, Babarskiene MR, Babeanu S, Bossini A, Gil-Extremera B, Girerd X, Laks T, Lilov E, Moisseyev V, Tuomilehto J, Vanhanen H, Webster J, Yodfat Y, Fagard R. Prevention of dementia in randomised double-blind placebo-controlled Systolic Hypertension in Europe (Syst-Eur) trial. Lancet. 1998 Oct 24;352(9137):1347-51. PMID: 9802273

13. Peters R, Beckett N, Forette F, Tuomilehto J, Clarke R, Ritchie C, Waldman A, Walton I, Poulter R, Ma S, Comsa M, Burch L, Fletcher A, Bulpitt C; HYVET investigators. Incident dementia and blood pressure lowering in the Hypertension in the Very Elderly Trial cognitive function assessment (HYVET-COG): a double-blind, placebo controlled trial. Lancet Neurol. 2008 Aug;7(8):683-9. PMID: 18614402

14. Tzourio C, Anderson C, Chapman N, Woodward M, Neal B, MacMahon S, Chalmers J. Effects of blood pressure lowering with perindopril and indapamide therapy on dementia

and cognitive decline in patients with cerebrovascular disease. Arch Intern Med 2003; 163(9): 1069-75.

15. Williamson JD, Pajewski NM, Auchus AP, Bryan RN, Chelune G, Cheung AK, Cleveland ML, Coker LH, Crowe MG, Cushman WC, Cutler JA, Davatzikos C, Desiderio L, Erus G, Fine LJ, Gaussoin SA, Harris D, Hsieh MK, Johnson KC, Kimmel PL, Tamura MK, Launer LJ, Lerner AJ, Lewis CE, Martindale-Adams J, Moy CS, Nasrallah IM, Nichols LO, Oparil S, Ogrocki PK, Rahman M, Rapp SR, Reboussin DM, Rocco MV, Sachs BC, Sink KM, Still CH, Supiano MA, Snyder JK, Wadley VG, Walker J, Weiner DE, Whelton PK, Wilson VM, Woolard N, Wright JT Jr, Wright CB. Effect of Intensive vs Standard Blood Pressure Control on Probable Dementia: A Randomized Clinical Trial. JAMA. 2019 Feb 12;321(6):553-561. PMID: 30688979

16. Goff DC, Jr., Lloyd-Jones DM, Bennett G, Coady S, D'Agostino RB, Sr., Gibbons R, Greenland P, Lackland DT, Levy D, O'Donnell CJ, Robinson JG, Schwartz JS, Shero ST, Smith SC, Jr., Sorlie P, Stone NJ, Wilson PWF. 2013 ACC/AHA guideline on the assessment of cardiovascular risk: a report of the American College of Cardiology/American Heart Association Task Force on Practice Guidelines. J Am Coll Cardiol. 2014;63(25 Pt B):2935-59. Epub 2013/11/19. doi: 10.1016/j.jacc.2013.11.005. PubMed PMID: 24239921; PMCID: PMC4700825.

17. He Y. Missing data analysis using multiple imputation: getting to the heart of the matter. Circulation Cardiovascular quality and outcomes. 2010;3(1):98-105. Epub 2010/02/04. doi: 10.1161/circoutcomes.109.875658. PubMed PMID: 20123676; PMCID: PMC2818781.

18. Sterne JA, White IR, Carlin JB, Spratt M, Royston P, Kenward MG, Wood AM, Carpenter JR. Multiple imputation for missing data in epidemiological and clinical research: potential and pitfalls. BMJ (Clinical research ed). 2009;338:b2393. Epub 2009/07/01. doi: 10.1136/bmj.b2393. PubMed PMID: 19564179; PMCID: PMC2714692.

19. Dunn OJ. Multiple Comparisons Among Means. J Am Stat Assoc. 1961;56(293):52–64.

20. Brown H, Prescott R. Applied Mixed Models in Medicine, 3rd Edition. New York: Wiley. 2015.

21. Diggle PJ, Liang KY, Zeger SL. Analysis of Longitudinal Data, 2nd Edition. Oxford: Oxford University Press, 2002.

22. Jung SH, Jeong JH. Rank tests for clustered survival data. Lifetime Data Anal. 2003;9(1):21-33. Epub 2003/02/27. doi: 10.1023/a:1021869803601. PubMed PMID: 12602772.

23. Cox DR. Regression models and life tables (with discussion). Journal of the Royal Statistical Society. 1972; Series B 34, 187-220. .

24. Lu SE, Wang MC. Marginal analysis for clustered failure time data. Lifetime Data Anal. 2005;11(1):61-79. Epub 2005/03/08. doi: 10.1007/s10985-004-5640-6. PubMed PMID: 15747590.

25. Bondarenko I, Raghunathan T. Graphical and numerical diagnostic tools to assess suitability of multiple imputations and imputation models. Stat Med. 2016;35(17):3007-20. Epub 2016/03/10. doi: 10.1002/sim.6926. PubMed PMID: 26952693.

26. Fitzmaurice GM, Lipsitz SR, Arriaga A, Sinha D, Greenberg C, Gawande AA. Almost efficient estimation of relative risk regression. Biostatistics. 2014;15(4):745-56.

27. Zou G. A modified Poisson regression approach to prospective studies with binary data. Am J Epidemiol. 2004;159(7):702-6.

28. Chen W, Qian L, Shi J, Franklin M. Comparing performance between log-binomial and robust Poisson regression models for estimating risk ratios under model misspecification. BMC Med Res Methodol. 2018;18, 63. https://doi.org/10.1186/s12874-018-0519-5.
