# Supplementary material for: Intensive Systolic Blood Pressure Reduction and Kidney and Cardiovascular Outcomes: A Secondary Analysis of a Randomized Clinical Trial
Source: JAMA Netw Open. 2025 Jul 11;8(7):e2519604. doi: 10.1001/jamanetworkopen.2025.19604 (PMC12254891; doi:10.1001/jamanetworkopen.2025.19604)
Supplement: Supplement 2. — eTable 1. Types of antihypertensive medications used during the 36-month follow-up among patients. eTable 2. Correlation analysis between BP decline and eGFR decline eFigure 1. Flow chart of subject inclusion eFigure 2. Cumulative incidence of stroke in the intervention versus usual care group eFigure 3. Cumulative incidence of myocardial infarction in the intervention versus usual care group eFigure 4. Cumulative incidence of heart failure in the intervention versus usual care group eFigure 5. Cumulative incidence of cardiovascular death in the intervention versus usual care group eFigure 6. Cumulative incidence of all-cause death in the intervention versus usual care group eFigure 7. Forest plots of subgroups for the composite cardiovascular outcome eFigure 8. Forest plots of subgroups for eGFR decline of ≥30% to a value < 60 ml/min/1.73m2 eFigure 9. Forest plots of subgroups for eGFR decline of ≥40% to a value < 60 ml/min/1.73m2 eFigure 10. Forest plots of subgroups for eGFR decline of ≥50% to a value < 60 ml/min/1.73m2 eFigure 11. Safety outcomes by randomization groups [file jamanetwopen-e2519604-s002.pdf]

# Supplemental Online Content

Sun G, Miao W, Liu S, et al. Intensive systolic blood pressure reduction and kidney and cardiovascular outcomes: a secondary analysis of a randomized clinical trial. *JAMA Netw Open*. 2025;8(7):e2519604. doi:10.1001/jamanetworkopen.2025.19604


**eTable 1.** Types of antihypertensive medications used during the 36-month follow-up among patients.

**eTable 2.** Correlation analysis between BP decline and eGFR decline

**eFigure 1.** Flow chart of subject inclusion

**eFigure 2.** Cumulative incidence of stroke in the intervention versus usual care group

**eFigure 3.** Cumulative incidence of myocardial infarction in the intervention versus usual care group

**eFigure 4.** Cumulative incidence of heart failure in the intervention versus usual care group

**eFigure 5.** Cumulative incidence of cardiovascular death in the intervention versus usual care group

**eFigure 6.** Cumulative incidence of all-cause death in the intervention versus usual care group

**eFigure 7.** Forest plots of subgroups for the composite cardiovascular outcome

**eFigure 8.** Forest plots of subgroups for eGFR decline of ≥30% to a value < 60 ml/min/1.73m$^2$

**eFigure 9.** Forest plots of subgroups for eGFR decline of ≥40% to a value < 60 ml/min/1.73m$^2$

**eFigure 10.** Forest plots of subgroups for eGFR decline of ≥50% to a value < 60 ml/min/1.73m$^2$

**eFigure 11.** Safety outcomes by randomization groups


This supplemental material has been provided by the authors to give readers additional information about their work.

© 2025 Sun G et al. *JAMA Network Open.*

## eTable 1. Types of antihypertensive medications used during the 36-month follow-up among patients.

| Antihypertensive medications | 60 ≤ eGFR <90 mL/min/1.73m² | | | eGFR ≥ 90 mL/min/1.73m² | | |
|---|---|---|---|---|---|---|
| | Intervention (N=3295) | Usual care (N=3234) | P value | Intervention (N=12109) | Usual care (N=11228) | P value |
| No. of participants who reported taking antihypertensive medications during the past 2 weeks | 3253(98.7) | 2645(81.8) | <0.001 | 11955(98.7) | 9146(81.5) | <0.001 |
| ACE inhibitors/angiotensin II receptor blockers | 2664(81.9) | 967(36.6) | <0.001 | 9757(80.6) | 3571(31.8) | <0.001 |
| Beta blockers | 36(1.1) | 50(1.9) | 0.108 | 156(1.3) | 155(1.4) | |
| Calcium channel blockers | 2963(91.1) | 1489(56.3) | <0.001 | 10585(87.4) | 4710(41.9) | <0.001 |
| Diuretics | 2140(65.8) | 286(10.8) | <0.001 | 7960(65.7) | 931(8.3) | <0.001 |
| Others | 24(0.7) | 268(10.1) | <0.001 | 65(0.5) | 943(8.4) | <0.001 |

Abbreviations: ACE = angiotensin-converting enzyme

Data are numbers (percentages). The sums of proportions are over 100% due to participants who took multiple antihypertensive medications.

**ACE inhibitors/angiotensin II receptor blockers:** Candesartan, Captopril, Enalapril, Fosinopril, Imidapril, Irbesartan, Lisinopril, Losartan, Olmesartan, Perindopril, Ramipril, Telmisartan, and Valsartan. **Beta blockers:** Arotinolol, Atenolol, Bisoprolol, Carvedilol, Labetalol, Metoprolol, and Propranolol. **Calcium channel blockers:** Amlodipine, Benidipine, Cinildipine, Diltiazem, Felodipine, Lacidipine, Lercanidipine, Nifedipine, Nimodipine, Nitrendipine, and Verapamil. Diuretics: Furosemide, Hydrochlorothiazide, Indapamide, Spirolactone, and Triamterene. **Others**: Beijing compound antihypertensive tablets (reserpine-dihydralazine-hydrochlorothiazide-triamterene), Compound reserpine tablets (reserpine-hydrochlorothiazide-dihydralazine-promethazine), Compound antihypertensive tablets (reserpine-dihydralazine-hydrochlorothiazide), Zhenju compound antihypertensive tablets (clonidine-hydrochlorothiazide), Compound triazine and rutinum tablets (reserpine-dihydralazine-hydrochlorothiazide), Compound bendazol hydrochlorothiazide capsules (reserpine-hydrochlorothiazide), Compound tetrazine reserpine tablets (reserpine-dihydralazine-hydrochlorothiazide), Compound apocynum (dihydralazine-hydrochlorothiazide), Reserpine, Dihydralazine, Clonidine, and Urapid.

© 2025 Sun G et al. *JAMA Network Open.*

## eTable 2 Correlation analysis between BP decline and eGFR decline

| | Point Estimate (95% CI)* | *P* value |
|---|---|---|
| **Correlation analysis between SBP decline and eGFR decline** | | |
| overall | 0.023 (0.018, 0.029) | <0.001 |
| 60 ⩽ eGFR <90 mL/min/1.73m$^2$ | 0.031 (0.017, 0.045) | <0.001 |
| eGFR ⩾ 90 mL/min/1.73m$^2$ | 0.022 (0.016, 0.027) | <0.001 |
| **Correlation analysis between DBP decline and eGFR decline** | | |
| overall | 0.020 (0.010, 0.031) | <0.001 |
| 60 ⩽ eGFR <90 mL/min/1.73m$^2$ | 0.042 (0.015, 0.070) | <0.001 |
| eGFR ⩾ 90 mL/min/1.73m$^2$ | 0.014 (0.004, 0.024) | 0.008 |

Abbreviations: SBP = systolic blood pressure; DBP = diastolic blood pressure; eGFR = estimated glomerular filtration rate.

[a] Additionally adjusted for group age, sex, cigarette smoking, use of antihypertensive medication, history of cardiovascular disease, and baseline systolic blood pressure, low-density lipoprotein cholesterol, and fasting plasma glucose.

© 2025 Sun G et al. *JAMA Network Open.*

## eFigure 1. Flow chart of subject inclusion

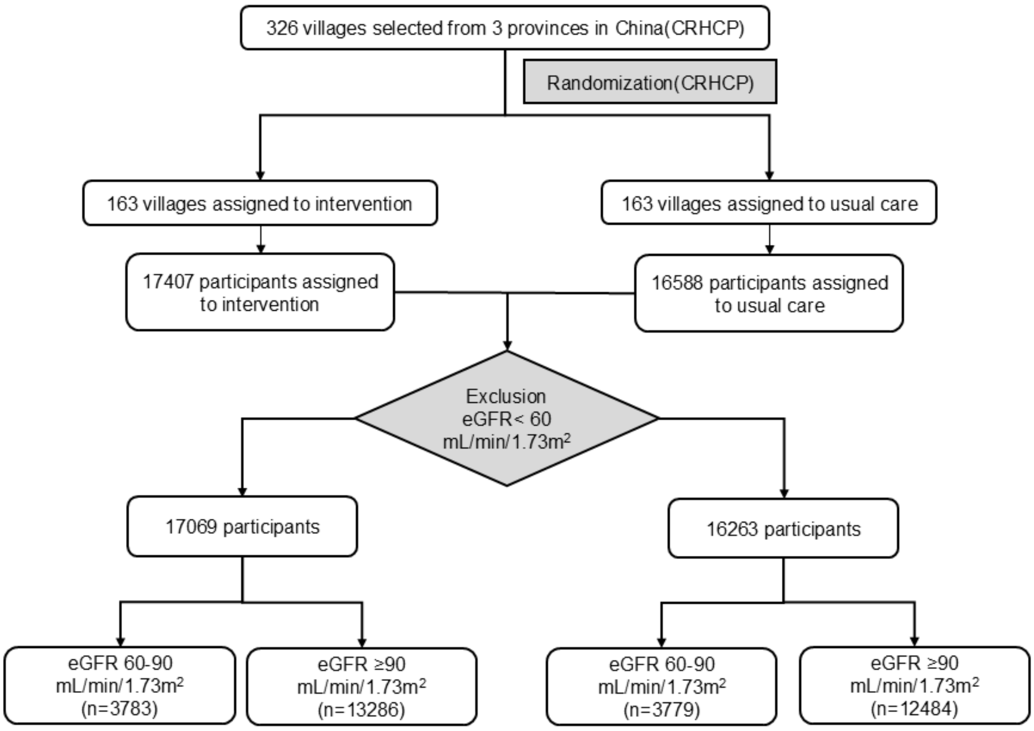


The randomization, recruitment, and enrollment of this study are shown.

Abbreviations: CRHCP = China Rural Hypertension Control Project; eGFR = estimated glomerular filtration rate

© 2025 Sun G et al. *JAMA Network Open.*

## eFigure 2. Cumulative incidence of stroke in the intervention versus usual care group.

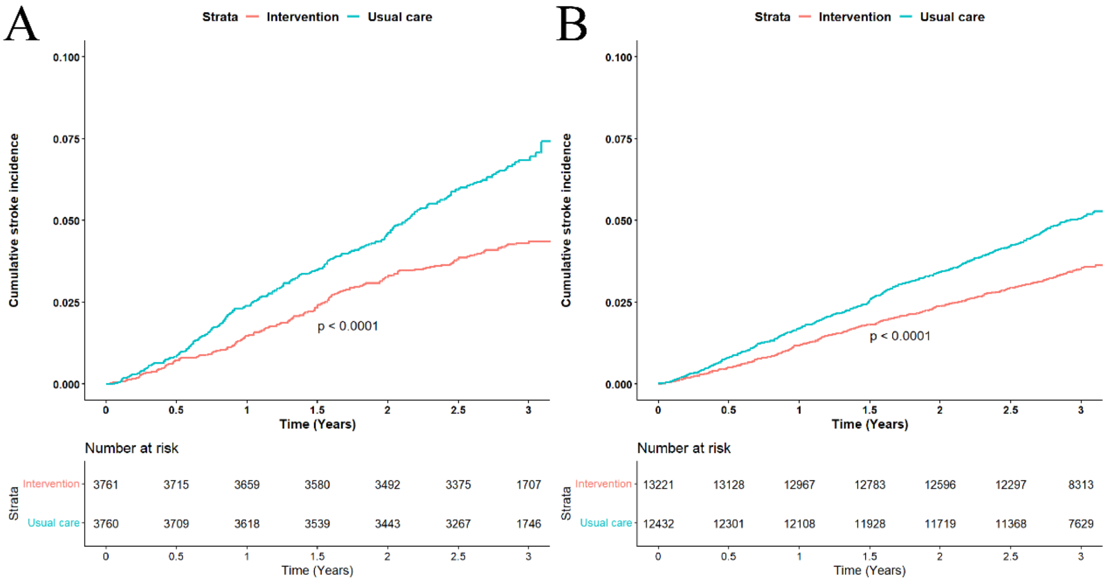


Cumulative incidence of stroke in participants with 60 ≤ eGFR＜90 mL/min/1.73m$^2$ (A), and participants with eGFR≥90 mL/min/1.73m$^2$ (B).

© 2025 Sun G et al. *JAMA Network Open.*

**eFigure 3. Cumulative incidence of myocardial infarction in the intervention versus usual care group.**

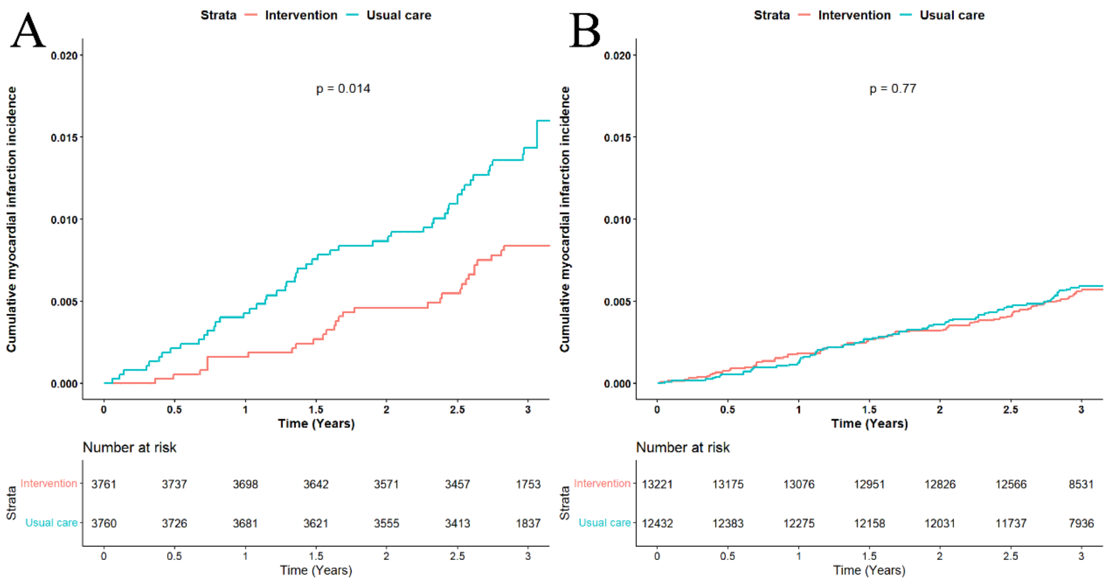


Cumulative incidence of myocardial infarction in participants with 60 ≤ eGFR＜90 mL/min/1.73m$^2$ (A), and participants with eGFR≥90 mL/min/1.73m$^2$ (B).

© 2025 Sun G et al. *JAMA Network Open.*

## eFigure 4. Cumulative incidence of heart failure in the intervention versus usual care group.

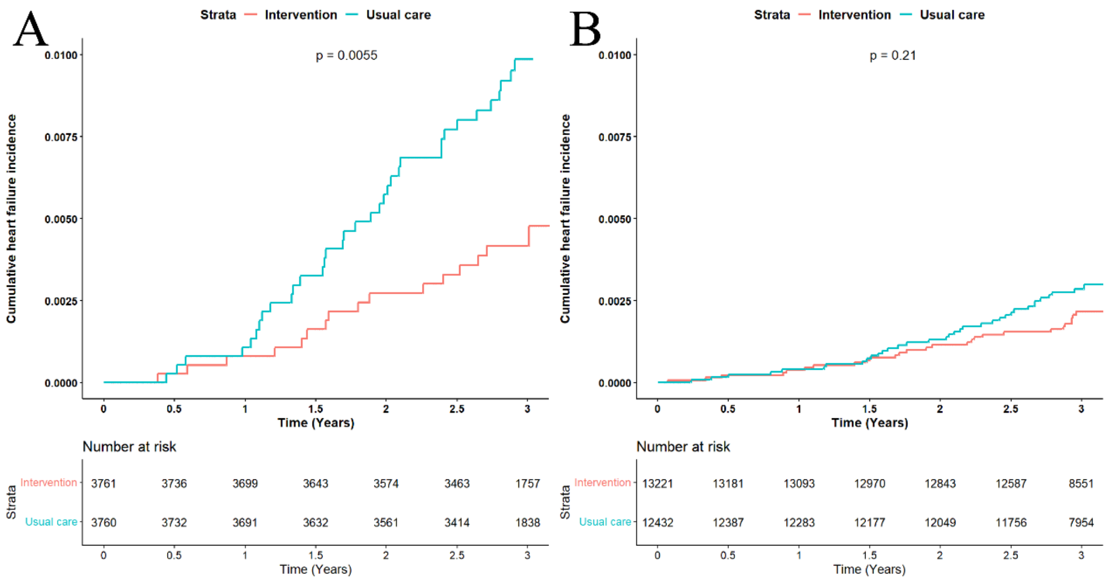


Cumulative incidence of heart failure in participants with 60 ≤ eGFR＜90 mL/min/1.73m$^2$ (A), and participants with eGFR≥90 mL/min/1.73m$^2$ (B).

© 2025 Sun G et al. *JAMA Network Open.*

## eFigure 5. Cumulative incidence of cardiovascular death in the intervention versus usual care group.

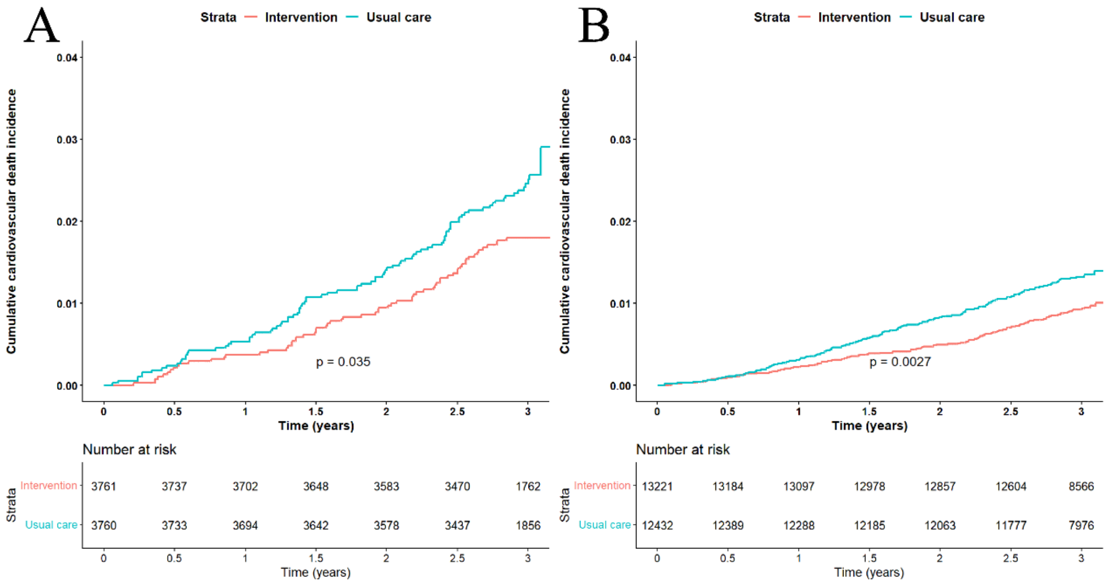


Cumulative incidence of cardiovascular death in participants with 60 ≤ eGFR＜90 mL/min/1.73m$^2$ (A), and participants with eGFR≥90 mL/min/1.73m$^2$ (B).

© 2025 Sun G et al. *JAMA Network Open.*

**eFigure 6. Cumulative incidence of all-cause death in the intervention versus usual care group.**

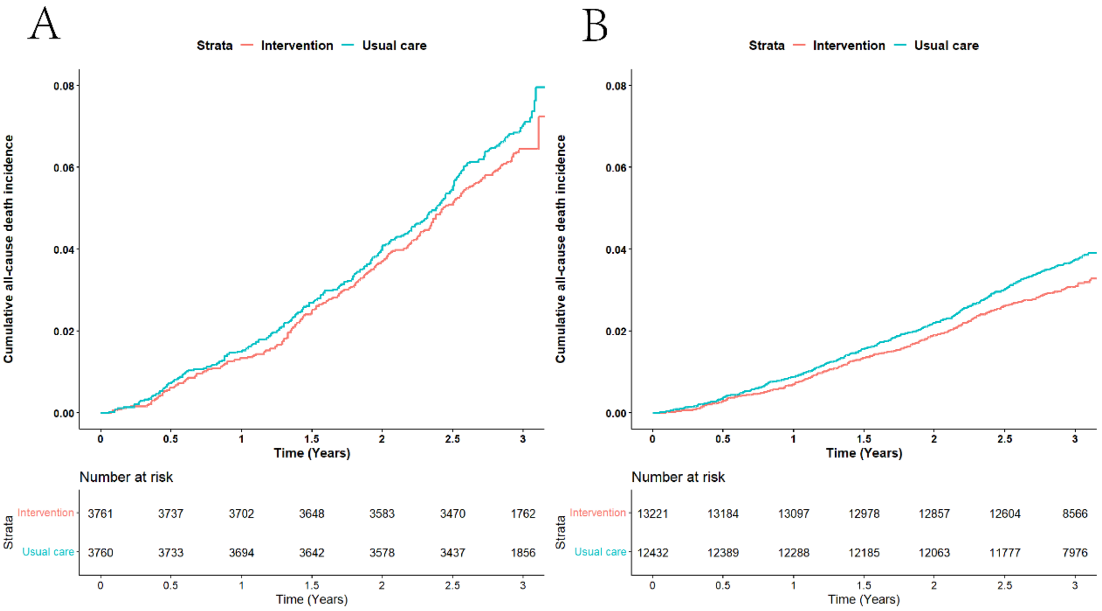


Cumulative incidence of all-cause death in participants with 60 ≤ eGFR < 90 mL/min/1.73m$^2$ (A), and participants with eGFR≥90 mL/min/1.73m$^2$ (B).

© 2025 Sun G et al. *JAMA Network Open.*

**eFigure 7. Forest plots of subgroups for the composite cardiovascular outcome.**

Participants with 60≤eGFR＜90mL/min/1.73m$^2$

Number of patients with total cardiovascular disease /total number (% per year)

| | Intervention | Usual care | Hazard ratio (95% CI) | *P* value | *P* for interaction |
|---|---|---|---|---|---|
| **Overall** | 210/3761(1.98) | 346/3760(3.32) | 0.58 (0.49, 0.70) | ＜0.001 | |
| **Sex** | | | | | |
| Male | 99/1377(2.59) | 167/1408(4.38) | 0.53 (0.40, 0.70) | ＜0.001 | 0.718 |
| Female | 111/2384(1.63) | 179/2352(2.71) | 0.59 (0.47, 0.74) | ＜0.001 | |
| **Age, years** | | | | | |
| <60 | 19/629(1.05) | 35/537(2.30) | 0.42 (0.22, 0.80) | 0.008 | 0.378 |
| ≥60 | 191/3132(2.17) | 311/3223(3.49) | 0.59 (0.49, 0.71) | ＜0.001 | |
| **Antihypertensive medication use** | | | | | |
| Treated | 139/2432(2.03) | 211/2161(3.52) | 0.57 (0.45, 0.71) | ＜0.001 | 0.856 |
| Untreated | 71/1329(1.89) | 135/1599(3.04) | 0.58 (0.44, 0.77) | ＜0.001 | |
| **History of CVD** | | | | | |
| Yes | 86/947(3.31) | 125/886(5.24) | 0.61 (0.45, 0.81) | ＜0.001 | 0.430 |
| No | 124/2814(1.55) | 221/2874(2.75) | 0.53 (0.42, 0.68) | ＜0.001 | |
| **History of DM** | | | | | |
| Yes | 38/397(3.51) | 44/371(4.36) | 0.73 (0.49, 1.08) | 0.113 | 0.127 |
| No | 172/3364(1.81) | 302/3389(3.21) | 0.54 (0.44, 0.67) | ＜0.001 | |

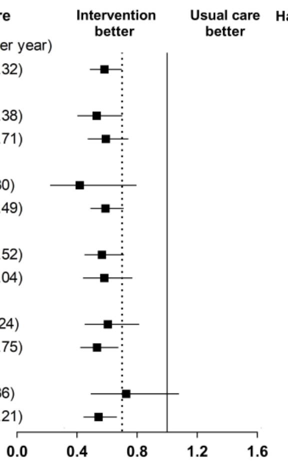


Participants with eGFR≥90mL/min/1.73m$^2$

Number of patients with total cardiovascular disease /total number (% per year)

| | Intervention | Usual care | Hazard ratio (95% CI) | *P* value | *P* for interaction |
|---|---|---|---|---|---|
| **Overall** | 567/13221(1.48) | 741/12432(2.08) | 0.70 (0.63, 0.78) | ＜0.001 | |
| **Sex** | | | | | |
| Male | 302/5244(2.01) | 372/4815(2.74) | 0.71 (0.62, 0.82) | ＜0.001 | 0.718 |
| Female | 265/7977(1.14) | 369/7617(1.67) | 0.64 (0.54, 0.74) | ＜0.001 | |
| **Age, years** | | | | | |
| <60 | 155/5410(0.98) | 212/4868(1.50) | 0.59 (0.48, 0.71) | ＜0.001 | 0.378 |
| ≥60 | 412/7811(0.83) | 529/7564(2.45) | 0.71 (0.63, 0.80) | ＜0.001 | |
| **Antihypertensive medication use** | | | | | |
| Treated | 367/7837(0.62) | 461/6561(2.46) | 0.64 (0.56, 0.73) | ＜0.001 | 0.856 |
| Untreated | 200/5384(0.28) | 280/5871(1.65) | 0.72 (0.60, 0.86) | ＜0.001 | |
| **History of CVD** | | | | | |
| Yes | 227/2639(0.06) | 300/2378(4.57) | 0.64 (0.56, 0.74) | ＜0.001 | 0.430 |
| No | 340/10582(0.10) | 441/10054(1.51) | 0.69 (0.60, 0.80) | ＜0.001 | |
| **History of DM** | | | | | |
| Yes | 67/1099(0.14) | 90/995(3.23) | 0.65 (0.47, 0.88) | 0.006 | 0.127 |
| No | 500/12122(0.42) | 651/11437(1.98) | 0.68 (0.60, 0.76) | ＜0.001 | |

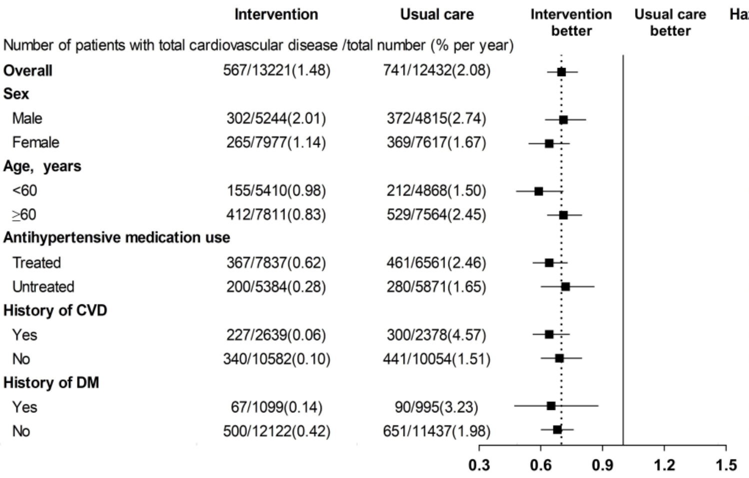


Abbreviations: eGFR=estimated glomerular filtration rate; CVD = cardiovascular disease. DM=diabetes.

Error bars indicate 95% Cis.

© 2025 Sun G et al. *JAMA Network Open.*

## eFigure 8. Forest plots of subgroups for eGFR decline of ≥30% to a value < 60 ml/min/1.73m$^2$.

**Participants with 60≤eGFR<90 mL/min/1.73m$^2$**

| | Intervention | Usual care | Intervention better | Usual care better | Rate ratio (95% CI) | *P* value | *P* for interaction |
|---|---|---|---|---|---|---|---|
| **eGFR decline of ≥30% to a value < 60 mL/min/1.73m$^2$** | | | | | | | |
| **Overall** | 121/3295 | 101/3234 | | | 1.17 (0.84, 1.62) | 0.356 | |
| **Sex** | | | | | | | |
| Male | 47/1167 | 32/1189 | | | 1.51 (0.89, 2.53) | 0.124 | 0.189 |
| Female | 74/2128 | 69/2045 | | | 1.02 (0.70, 1.51) | 0.898 | |
| **Age, years** | | | | | | | |
| <60 | 16/575 | 15/476 | | | 0.88 (0.44, 1.79) | 0.729 | 0.383 |
| ≥60 | 105/2720 | 86/2758 | | | 1.25 (0.89, 1.76) | 0.201 | |
| **Antihypertensive medication use** | | | | | | | |
| Treated | 80/2152 | 54/1879 | | | 1.36 (0..89, 2.07) | 0.156 | 0.419 |
| Untreated | 41/1143 | 47/1355 | | | 1.01 (0.64, 1.62) | 0.951 | |
| **History of CVD** | | | | | | | |
| Yes | 35/809 | 29/755 | | | 1.10 (0.67, 1.80) | 0.707 | 0.764 |
| No | 86/2486 | 72/2479 | | | 1.20 (0.82, 1.74) | 0.346 | |
| **History of DM** | | | | | | | |
| Yes | 15/336 | 14/313 | | | 0.85 (0.45, 1.60) | 0.605 | 0.182 |
| No | 106/2959 | 87/2921 | | | 1.31 (0.90, 1.92) | 0.163 | |

0.1 1 10

**Participants with eGFR≥90 mL/min/1.73m$^2$**

| | Intervention | Usual care | Intervention better | Usual care better | Rate ratio (95% CI) | *P* value | *P* for interaction |
|---|---|---|---|---|---|---|---|
| **eGFR decline of ≥30% to a value < 60 mL/min/1.73m$^2$** | | | | | | | |
| **Overall** | 107/12109 | 82/11228 | | | 1.21 (0.85, 1.73) | 0.288 | |
| **Sex** | | | | | | | |
| Male | 40/4711 | 22/4262 | | | 1.64 (0.90, 2.98) | 0.104 | 0.161 |
| Female | 67/7398 | 60/6966 | | | 1.02 (0.63, 1.63) | 0.949 | |
| **Age, years** | | | | | | | |
| <60 | 23/4988 | 16/4443 | | | 1.29 (0.58, 2.89) | 0.534 | 0.881 |
| ≥60 | 84/7121 | 66/6785 | | | 1.19 (0.81, 1.74) | 0.387 | |
| **Antihypertensive medication use** | | | | | | | |
| Treated | 74/7212 | 44/5951 | | | 1.37 (0.90, 2.08) | 0.141 | 0.196 |
| Untreated | 33/4897 | 38/5277 | | | 0.94 (0.53, 1.66) | 0.833 | |
| **History of CVD** | | | | | | | |
| Yes | 25/2352 | 19/2099 | | | 1.29 (0.71, 2.35) | 0.411 | 0.813 |
| No | 82/9757 | 63/9129 | | | 1.21 (0.80, 1.84) | 0.359 | |
| **History of DM** | | | | | | | |
| Yes | 17/996 | 13/875 | | | 1.15 (0.57, 2.29) | 0.696 | 0.837 |
| No | 90/11113 | 69/10353 | | | 1.24 (0.84, 1.84) | 0.279 | |

0.1 1 10

Abbreviations: eGFR=estimated glomerular filtration rate; CVD = cardiovascular disease. DM= diabetes mellitus

Error bars indicate 95% CIs.

© 2025 Sun G et al. *JAMA Network Open.*

## eFigure 9. Forest plots of subgroups for eGFR decline of ≥40% to a value < 60 ml/min/1.73m$^2$.

**Participants with 60≤eGFR<90 mL/min/1.73m$^2$**

| | Intervention | Usual care | Intervention better / Usual care better | Rate ratio (95% CI) | *P* value | *P* for interaction |
|---|---|---|---|---|---|---|
| **eGFR decline of ≥40% to a value < 60 mL/min/1.73m$^2$** | | | | | | |
| **Overall** | 49/3295 | 33/3234 | | 1.46 (0.86, 2.49) | 0.159 | |
| **Sex** | | | | | | |
| Male | 18/1167 | 6/1189 | | 3.06 (1.21, 7.71) | 0.018 | 0.059 |
| Female | 31/2128 | 27/2045 | | 1.10 (0.55, 2.19) | 0.793 | |
| **Age, years** | | | | | | |
| <60 | 6/575 | 5/476 | | 0.99 (0.30, 3.26) | 0.991 | 0.490 |
| ≥60 | 43/2720 | 28/2758 | | 1.58 (0.91, 2.74) | 0.107 | |
| **Antihypertensive medication use** | | | | | | |
| Treated | 30/2152 | 17/1879 | | 1.62 (0.76, 3.42) | 0.209 | 0.844 |
| Untreated | 19/1143 | 16/1355 | | 1.41 (0.72, 2.74) | 0.312 | |
| **History of CVD** | | | | | | |
| Yes | 16/809 | 13/755 | | 1.36 (0.,63, 2.94) | 0.43 | 0.847 |
| No | 33/2486 | 20/2479 | | 1.57 (0.80, 3.10) | 0.188 | |
| **History of DM** | | | | | | |
| Yes | 7/336 | 9/313 | | 1.04 (0.48, 2.28) | 0.918 | 0.318 |
| No | 42/2959 | 24/2921 | | 1.76 (0.90, 3.43) | 0.097 | |

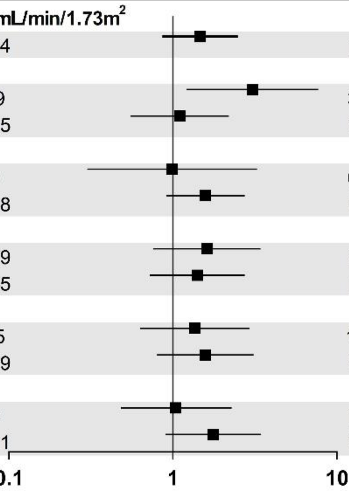


**Participants with eGFR≥90 mL/min/1.73m$^2$**

| | Intervention | Usual care | Intervention better / Usual care better | Rate ratio (95% CI) | *P* value | *P* for interaction |
|---|---|---|---|---|---|---|
| **eGFR decline of ≥40% to a value < 60 mL/min/1.73m$^2$** | | | | | | |
| **Overall** | 80/12109 | 72/11228 | | 1.04 (0.70, 1.56) | 0.829 | |
| **Sex** | | | | | | |
| Male | 31/4711 | 19/4262 | | 1.49 (0.75, 2.93) | 0.257 | 0.134 |
| Female | 49/7398 | 53/6966 | | 0.85 (0.50, 1.44) | 0.537 | |
| **Age, years** | | | | | | |
| <60 | 21/4988 | 16/4443 | | 1.18 (0.50, 2.79) | 0.704 | 0.689 |
| ≥60 | 59/7121 | 56/6785 | | 0.98 (0.62, 1.54) | 0.927 | |
| **Antihypertensive medication use** | | | | | | |
| Treated | 55/7212 | 35/5951 | | 1.30 (0.80, 2.10) | 0.283 | 0.087 |
| Untreated | 25/4897 | 37/5277 | | 0.74 (0.40, 1.38) | 0.346 | |
| **History of CVD** | | | | | | |
| Yes | 18/2352 | 14/2099 | | 1.36 (0.55, 3.36) | 0.501 | 0.462 |
| No | 62/9757 | 58/9129 | | 1.00 (0.64, 1.58) | 0.970 | |
| **History of DM** | | | | | | |
| Yes | 14/996 | 11/875 | | 1.13 (0.61, 2.11) | 0.693 | 0.727 |
| No | 66/11113 | 61/10353 | | 1.01 (0.64, 1.60) | 0.964 | |

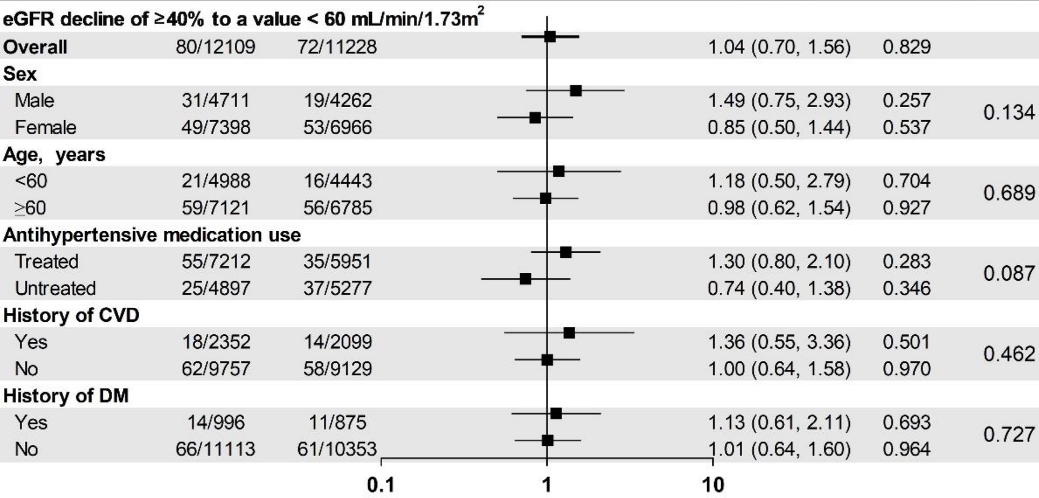


Abbreviations: eGFR: estimated glomerular filtration rate; CVD = cardiovascular disease. DM= diabetes mellitus

Error bars indicate 95% CIs.

© 2025 Sun G et al. *JAMA Network Open.*

## eFigure 10. Forest plots of subgroups for eGFR decline of ≥50% to a value < 60 ml/min/1.73m$^2$.

**Participants with 60≤eGFR<90 mL/min/1.73m$^2$**

| | Intervention | Usual care | Rate ratio (95% CI) | *P* value | *P* for interaction |
|---|---|---|---|---|---|
| **eGFR decline of ≥50% to a value < 60 mL/min/1.73m$^2$** | | | | | |
| **Overall** | 25/3295 | 16/3234 | 1.69 (0.70, 4.11) | 0.246 | |
| **Sex** | | | | | |
| Male | 9/1167 | 2/1189 | 4.59 (0.99, 21.23) | 0.051 | 0.098 |
| Female | 16/2128 | 14/2045 | 1.25 (0.25, 6.21) | 0.786 | |
| **Age, years** | | | | | |
| <60 | 3/575 | 1/476 | 2.48 (0.26, 23.88) | 0.431 | 0.670 |
| ≥60 | 22/2720 | 15/2758 | 1.60 (0.63, 4.06) | 0.321 | |
| **Antihypertensive medication use** | | | | | |
| Treated | 15/2151 | 8/1879 | 2.08 (0.30, 14.50) | 0.459 | 0.879 |
| Untreated | 10/1143 | 8/1355 | 1.50 (0.21, 10.80) | 0.689 | |
| **History of CVD** | | | | | |
| Yes | 8/809 | 5/755 | 1.87 (0.56, 6.22) | 0.305 | 0.694 |
| No | 11/2479 | 17/2486 | 1.64 (0.35, 7.65) | 0.531 | |
| **History of DM** | | | | | |
| Yes | 3/336 | 5/313 | 0.64 (0.18, 2.27) | 0.491 | 0.118 |
| No | 22/2959 | 11/2921 | 2.48 (2.47, 2.49) | <0.001 | |

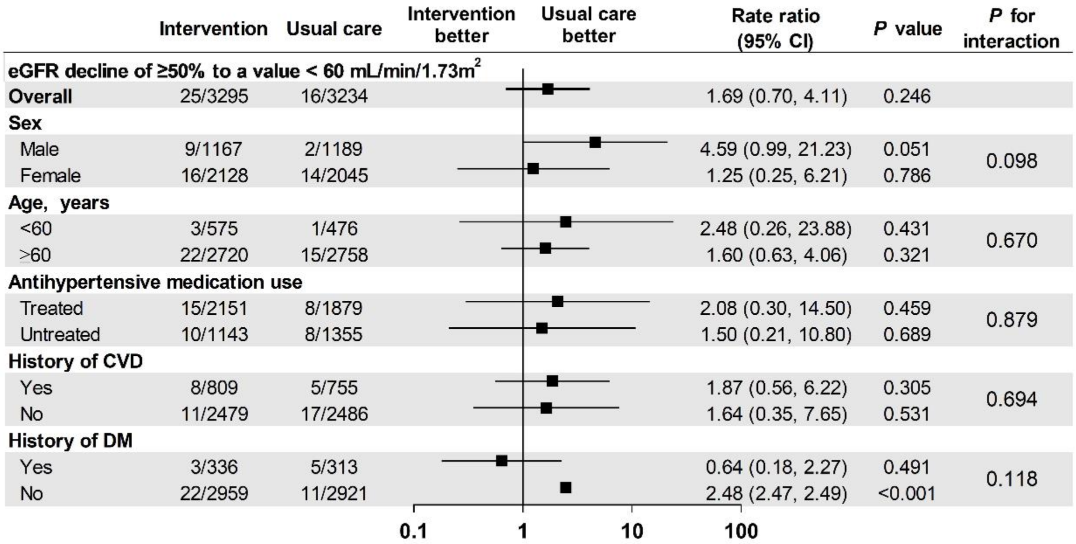


**Participants with eGFR≥90 mL/min/1.73m$^2$**

| | Intervention | Usual care | Rate ratio (95% CI) | *P* value | *P* for interaction |
|---|---|---|---|---|---|
| **eGFR decline of ≥50% to a value < 60 mL/min/1.73m$^2$** | | | | | |
| **Overall** | 39/12109 | 29/11228 | 1.30 (0.71, 2.38) | 0.392 | |
| **Sex** | | | | | |
| Male | 19/4711 | 7/4262 | 2.46 (1.03, 5.84) | 0.042 | 0.051 |
| Female | 20/7398 | 22/6966 | 0.88 (0.35, 2.21) | 0.779 | |
| **Age, years** | | | | | |
| <60 | 14/4988 | 9/4443 | 1.36 (0.23, 7.94) | 0.733 | 0.772 |
| ≥60 | 25/7121 | 20/6785 | 1.17 (0.58, 2.38) | 0.661 | |
| **Antihypertensive medication use** | | | | | |
| Treated | 28/7212 | 15/5951 | 1.57 (0.73, 3.39) | 0.247 | 0.244 |
| Untreated | 11/4897 | 14/5277 | 0.94 (0.18, 4.80) | 0.938 | |
| **History of CVD** | | | | | |
| Yes | 11/2352 | 4/2099 | 2.45 (0.78, 7.69) | 0.125 | 0.189 |
| No | 28/9757 | 25/9129 | 1.14 (0.56, 2.32) | 0.726 | |
| **History of DM** | | | | | |
| Yes | 6/996 | 7/875 | 1.02 (0.43, 2.40) | 0.963 | 0.580 |
| No | 33/11113 | 22/10353 | 1.43 (0.67, 3.06) | 0.350 | |

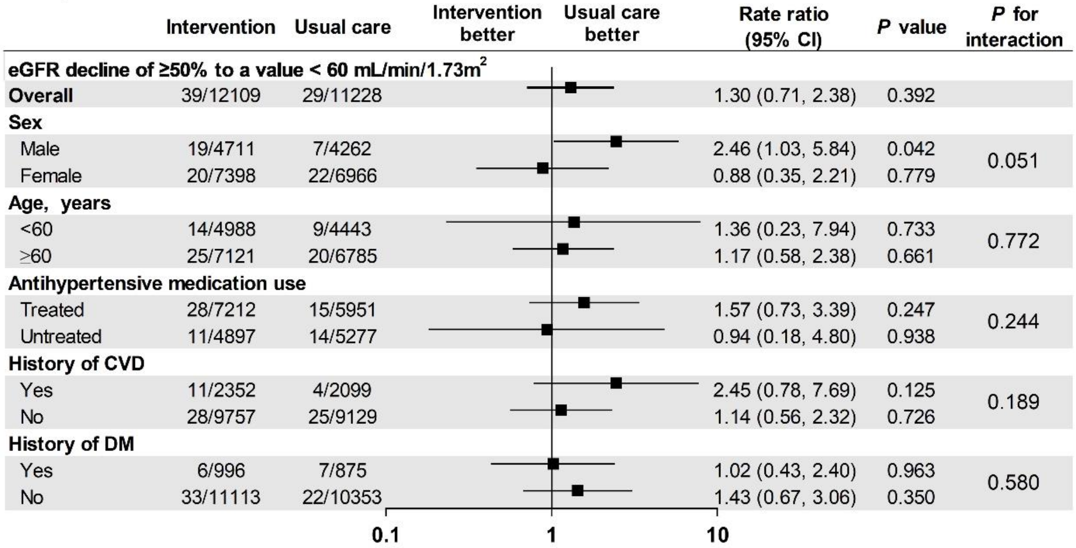


Abbreviations: eGFR: estimated glomerular filtration rate; CVD = cardiovascular disease. DM= diabetes mellitus

Error bars indicate 95% CIs.

© 2025 Sun G et al. *JAMA Network Open.*

## eFigure 11. Safety outcomes by randomization groups.

| Variables | Intervention | Usual care | Risk Ratio (95% CI) | *P* value |
|---|---|---|---|---|
| **A. Participants with 60 ≤ eGFR<90 mL/min/1.73m²** | | | | |
| Injurious falls | 16/3783 | 23/3779 | 0.69 (0.34, 1.37) | 0.267 |
| Hypotension | 61/3783 | 31/3779 | 1.97 (1.26, 3.13) | 0.002 |
| Symptomatic hypotension | 26/3783 | 24/3779 | 1.08 (0.60, 1.97) | 0.783 |
| Syncope | 20/3783 | 20/3779 | 1.00 (0.51, 1.96) | 0.997 |
| Electrolyte at 36 months | | | | |
| Serum sodium < 130 mmol/L | 1/3295 | 2/3234 | 0.49(0.01, 9.43) | 0.615 |
| Serum sodium > 150 mmol/L | 7/3295 | 21/3234 | 0.33 (0.12, 0.80) | 0.007 |
| Serum potassium < 3.0 mmol/L | 10/3295 | 2/3234 | 4.91 (1.05, 46.06) | 0.024 |
| Serum potassium > 5.5 mmol/L | 28/3295 | 31/3234 | 0.89 (0.51, 1.53) | 0.647 |
| **B. Participants with eGFR≥90 mL/min/1.73m²** | | | | |
| Injurious falls | 77/13286 | 59/12484 | 1.23 (0.86, 1.75) | 0.239 |
| Hypotension | 239/13286 | 113/12484 | 1.99 (1.58, 2.51) | <0.001 |
| Symptomatic hypotension | 98/13286 | 91/12484 | 1.01 (0.75, 1.36) | 0.936 |
| Syncope | 50/13286 | 42/12484 | 1.12 (0.73, 1.73) | 0.595 |
| Electrolyte at 36 months | | | | |
| Serum sodium < 130 mmol/L | 6/12111 | 4/11230 | 1.39 (0.33, 6.70) | 0.629 |
| Serum sodium > 150 mmol/L | 71/12111 | 219/11230 | 0.30 (0.23, 0.39) | <0.001 |
| Serum potassium < 3.0 mmol/L | 25/12110 | 16/11230 | 1.15 (0.74, 2.90) | 0.249 |
| Serum potassium > 5.5 mmol/L | 48/12110 | 55/11230 | 0.81 (0.54, 1.21) | 0.286 |

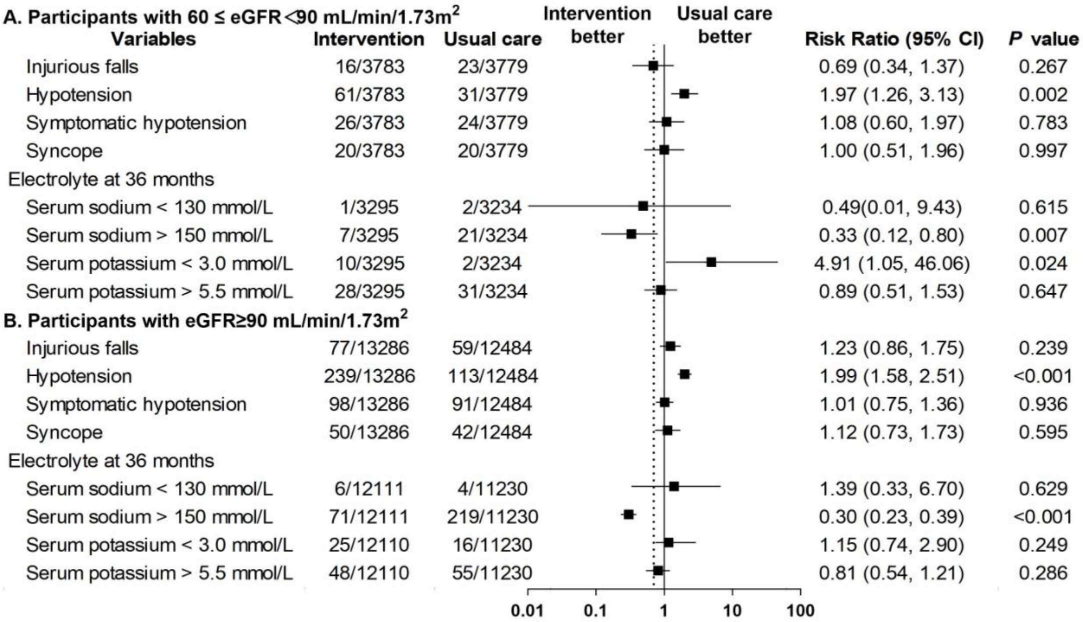


The occurrence of adverse reactions during intensive blood pressure in the intervention and usual care groups. The population involved participants with 60≤eGFR<90 mL/min/1.73m² (A). The population involved participants with eGFR≥90 mL/min/1.73m² (B).

Abbreviations: CI = confidence interval, eGFR: estimated glomerular filtration rate.

Notes: Injurious falls was self-reported and defined as a fall that resulted in seeking medical care. Hypotension was defined as systolic blood pressure <90 mmHg at a village doctor visit or a study data a collection visit at months 6, 12, 18, 24, 30, and 36. Symptomatic hypotension was self-reported and confirmed by systolic blood pressure <90 mmHg a at a village doctor visit. Syncope was defined as self-reported temporary loss of consciousness that resulted in seeking medical care.

© 2025 Sun G et al. *JAMA Network Open.*
