# Supplementary material for: Intensive Systolic Blood Pressure Reduction and Kidney and Cardiovascular Outcomes: A Secondary Analysis of a Randomized Clinical Trial
Source: JAMA Netw Open. 2025 Jul 11;8(7):e2519604. doi: 10.1001/jamanetworkopen.2025.19604 (PMC12254891; doi:10.1001/jamanetworkopen.2025.19604)
Supplement: Supplement 3. — Data Sharing Statement [file jamanetwopen-e2519604-s003.pdf]

Data Sharing Statement

Sun. Intensive Systolic Blood Pressure Reduction and Kidney and Cardiovascular Outcomes. *JAMA Netw Open*. Published July 09, 2025. doi:10.1001/jamanetworkopen.2025.19604

Data
Additional Information: The trial is registered with ClinicalTrials.gov, NCT03527719.
Data available: Yes
Data types: Deidentified participant data, Data dictionary
How to access data: Data from this study can be requested from Prof Yingxian Sun (yxsun@cmu.edu.cn)
When available: With publication

Supporting Documents
Document types: None

Additional Information
Who can access the data: Specific requests for data will require the submission of a proposal with a valuable research question as assessed by the study steering committee and require a data access agreement to be signed.
Types of analyses: Any analyses with a valuable research question
Mechanisms of data availability: After approval of a proposal as assessed by the study steering committee and with a signed data access agreement.
